# Dual diagnosis of acidification dynamics in Amazonian urban lakes: multivariate analysis and calcite saturation index for status and vulnerability assessment

**DOI:** 10.1007/s10661-026-15475-y

**Published:** 2026-05-27

**Authors:** Carlos Noriega, Cryssia Romão, Rafael Aquino, Sury Monteiro, Bruna Moraes, Rodrigo Brito, Carmen Medeiros, Marcelo Rollnic, Moacyr Araujo

**Affiliations:** 1 Department of Oceanography, Federal University of Pernambuco – UFPE, Av. Arquitetura S/N, Recife, 50740-550 Brazil; 2 Laboratório de Pesquisa E Monitoramento Ambiental Marinho (LAPMAR), Federal University of Pará, Rua Augusto Corrêa N° 1, Belém, PA 66075-900 Brazil; 3 Brazilian Research Network On Global Climate Change (Rede CLIMA), Av. Dos Astronautas, São José Dos Campos, SP 1758, 01227-010 Brazil

**Keywords:** Water quality, Acidification index, Limnology, Multivariate analysis, Tropical lakes

## Abstract

This study presents a dual diagnosis of acidification processes in the lakes in Lakes Água Preta (AP) and Bolonha (BL), two strategic reservoirs supplying the metropolitan area of Belém, Eastern Amazon (Brazil). We assessed the status and vulnerability of these ecosystems by integrating multivariate statistical analysis (PCA) with the Acidification Sensitivity Index (ASI). Monthly monitoring was conducted between September 2023 and August 2024, evaluating 21 parameters across the surface and bottom layers. Results indicate that both systems are chronically sensitive, with annual mean ASI values of -1.03 (AP) and -1.10 (BL), reflecting low alkaline reserves. Principal Component Analysis (PCA) revealed that Lake AP exhibits greater metabolic instability, with a score amplitude of 9.95 compared to 7.72 for BL. During the dry season, stress was predominantly biogenic; dissolved CO_2_ reached 317.0 μmoles L^−1^, accompanied by frequent bottom anoxia, with 62.0% of Dissolved Oxygen (DO) samples falling below the legal limit of 5.0 mg L^−1^. Conversely, during the wet season, vulnerability was exacerbated by ionic dilution, as Total Alkalinity (TA) dropped to critical levels (39.0–48.0 μmoles L^−1^) and the ASI reached a minimum of -2.57. While Lake AP is primarily governed by the inorganic carbon cycle, BL is driven by cultural eutrophication. This study concludes that stand-alone pH monitoring is insufficient to capture the thermodynamic complexity of these tropical reservoirs. These findings provide novel scientific evidence for the necessity of seasonally differentiated management to ensure water security in urban-impacted Amazonian systems.

**Supplementary Information:**

The online version contains supplementary material available at 10.1007/s10661-026-15475-y.

## Introduction

The integrity of freshwater ecosystems worldwide is under increasing threat from rapid urban expansion and shifting land-use patterns within watersheds. This landscape of unregulated anthropogenic occupation has become the primary driver of cultural eutrophication process that degrades water quality by elevating nutrient concentrations and drastically depleting local biodiversity (Callisto et al., 2014; Suresh et al., 2023; Zheng et al., 2021). Within urban lentic systems, low water turnover rates further constrain self-purification capacity, rendering these environments highly susceptible to pollutant accumulation from both external runoff and the internal release of nutrients from bottom sediments (Yang et al., 2020). In tropical regions, the challenges facing urban lakes take on even more critical dimensions. Unlike temperate systems, tropical lakes are subject to high metabolic rates year-round, as the combination of elevated temperatures and intense solar radiation accelerates nutrient cycling. In these latitudes, acidification emerges as a complex, predominantly biogenic phenomenon, contrasting with the atmospheric deposition-driven acidification common in the Northern Hemisphere (Fölster et al., 2014). The tropical metabolic dynamic, characterized by intense biomass decomposition, generates a constant supersaturation of dissolved carbon dioxide (CO_2_), which acts as the chemical engine of acidity in these water bodies (Silva et al., 2022; Cole & Prairie, 2009). Furthermore, surrounding vegetation types and the seasonal exposure of littoral areas during water level fluctuations can intensify gas emissions, significantly altering the acid–base balance (Almeida et al., 2019). To establish a precise geochemical health diagnosis of such water bodies, it was fundamental to analyze a broad spectrum of physicochemical variables. The equilibrium between parameters such as alkaline reserve, ionic conductivity, and the presence of metals defines the resilience or vulnerability of a lake to acidic shocks (Bishop, 1997). However, the complexity of these interactions in stratified tropical systems necessitated the use of methods capable of processing multiple variables simultaneously, transcending the limitations of isolated pH measurements. In this regard, the application of multivariate statistical techniques proved effective in identifying key environmental stressors and chemical variability patterns in environments under anthropogenic pressure (Silva et al., 2022). Complementarily, the use of ionic sensitivity indices allowed for an assessment that distinguished the current state of acidity from the system's structural vulnerability. The objective of this study was to perform a dual diagnosis of acidification dynamics in the Bolonha (LB) and Água Preta (AP) lakes, two strategic urban reservoirs located in the Eastern Amazon. These lakes represent a typical scenario of tropical reservoirs impacted by urbanization, where pressure from both the forest environment and the urban landscape shapes water chemistry. By integrating multivariate analyses and calculating mineral saturation indices, this study aimed to assess acidification dynamics and ecosystem vulnerability to seasonal variability and anthropogenic pressures, providing a scientific basis for regional water resource management.

## Material and methods

### Study area

**Fig. 1:**
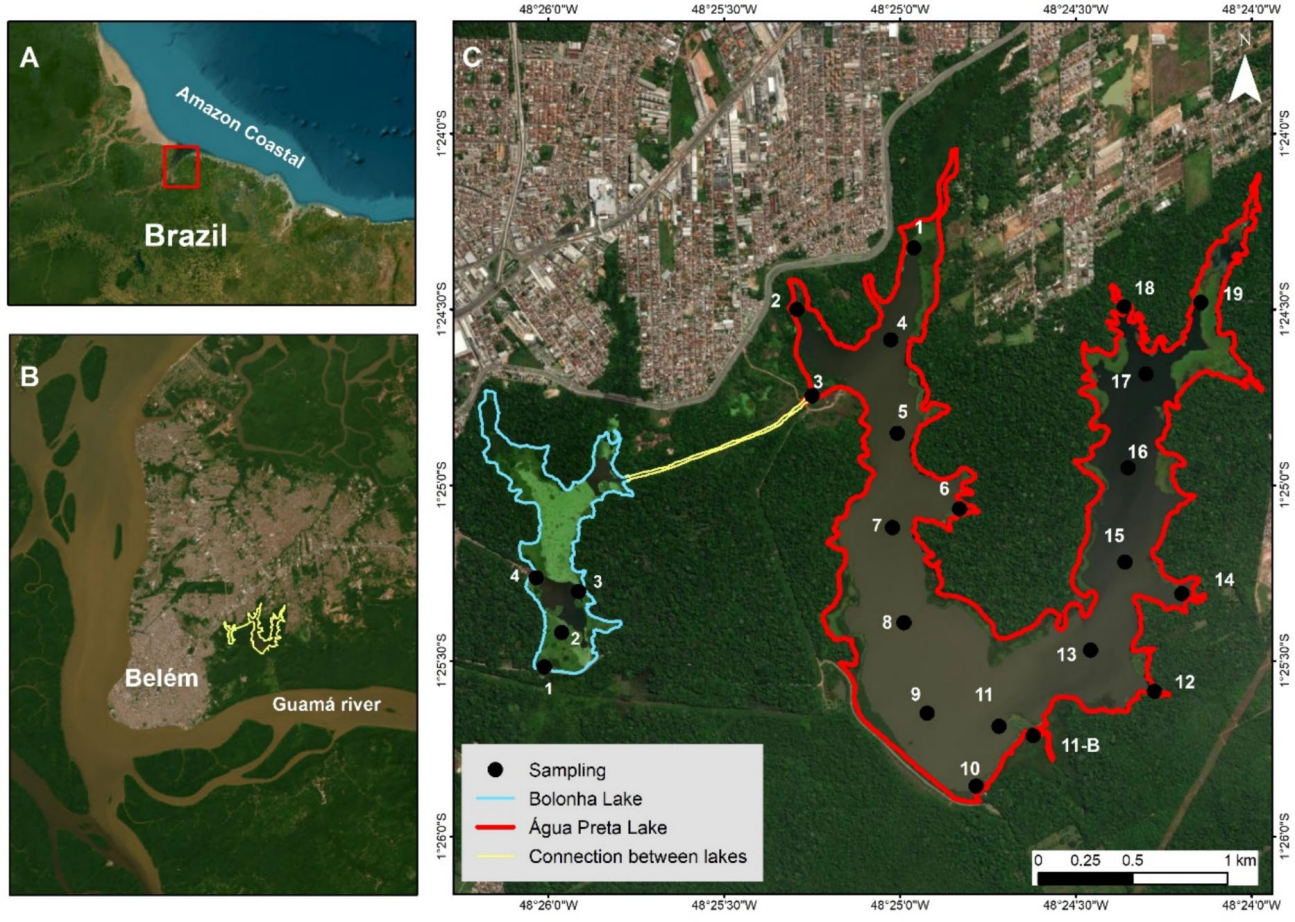
Location of the study area in the Água Preta and Bolonha lakes in thecity of Belém, Pará, Brazil. The red dots indicate the sampling stations for the 2023–2024 study. Source: Google Earth,2026

Lakes Bolonha (577,000 m^2^) and Água Preta (3,116,000 m^2^) are situated within the Utinga watershed in Belém, Northern Brazil (Silva et al., 2022). These water bodies serve as the primary reservoirs supplying drinking water to approximately 2 million residents in Belém and neighboring municipalities (Fig. 1). Bathymetric data indicate an average depth of 3.0 m, with negligible seasonal fluctuations in water level (Fig. S1; Supplementary Material). The lakes are fed by the Guamá River via an intake channel with an estimated discharge of approximately 7.0 m^3^ s^−1^ (Silva et al., 2022). The Guamá River, a typical Amazonian system, is classified as a clearwater river (Junk et al., 2011). Its waters are characterized by circum-neutral pH (6.0–8.5) and moderate ionic content, with electrical conductivity typically ranging from ~ 30 to 100 µS cm^−1^, consistent with values reported for Amazonian clearwater systems (Junk et al., 2011; Ríos-Villamizar et al., 2020; Stallard & Edmond, 1983). The predominant soil type in the Água Preta and Bolonha catchments is Latosol (Ferralsol), covering approximately 85% of the surrounding landscape (Junk et al., 2011). The regional climate is hot and humid, classified as *Af* (tropical rainforest) according to the Köppen system. The average annual precipitation is approximately 2,800 mm, with the highest rainfall occurring between March and May. Between September 2023 and August 2024, twelve consecutive monthly monitoring campaigns were conducted in both lakes. These climatic periods are typical of the study region (Silva et al., 2022).

### Sampling and analytical methodologies

#### Field procedures and in situ monitoring

A comprehensive monthly monitoring program was implemented between September 2023 and August 2024, involving 30 sampling stations distributed across both reservoirs. At each site, water samples were retrieved from the surface and bottom layers, generating a total of 720 samples throughout the annual cycle. Sample collection was performed utilizing a 2-L Van Dorn sampler. The monitoring design encompassed the main phases of the regional hydrological cycle, including the dry season (September–November), the dry-to-wet transition (December–February), the wet season (March–May), and the wet-to-dry transition (June–August). These climatic periods are typical of the study region (Silva et al., 2022). Key physicochemical parameters—including temperature, pH, dissolved oxygen (DO), electrical conductivity (EC), turbidity, and total dissolved solids (TDS)—were quantified in situ using a HORIBA U-52 multiparameter probe. The measurement accuracy was ± 1% for EC and approximately ± 2% for TDS, the latter estimated from electrical conductivity using an internal conversion factor. The instrument was calibrated to provide high-resolution data, achieving accuracies of ± 0.3 °C for temperature, ± 0.1 units for pH, and ± 0.2 mg L^−1^ for DO. DO concentrations were converted from mg l^−1^ to μmol l^−1^ to standardize units across all carbonate system parameters (e.g., alkalinity and CO_2_), facilitating direct stoichiometric comparisons and biogeochemical analysis. The conversion followed the molar mass of O_2_ (31.998 g mol^−1^).

#### Sample processing and laboratory protocols

Water samples were initially stored in 1-L high-density polyethylene (HDPE) bottles, which had been pre-rinsed with deionized water. For specialized chemical analyses, aliquots were transferred to bottles previously decontaminated in a 10% (v/v) HCl bath for 24 h and thoroughly rinsed. Samples underwent filtration through 0.7 μm GF/F glass fiber membranes and were immediately cryopreserved at −20 °C.

Chlorophyll-*a* (Chl-a) concentrations were determined spectrophotometrically following the protocols established by Arar (1997). Dissolved inorganic nutrients (NH_4_^+^, NO_3_^−^, NO_2_^−^, PO_4_^3−^, and SiO_2_) were analyzed in triplicate according to Grasshoff et al. (1983) using a Quimis® Q898DRM spectrophotometer. Dissolved inorganic nitrogen (DIN) was calculated as the stoichiometric sum of nitrate, nitrite, and ammonium. Total alkalinity (TA) was quantified via inflection point titration, adhering to the U.S. Geological Survey (USGS) standards, yielding an analytical precision of 6 μmol L^−1^. Biochemical oxygen demand (BOD_5_) was assessed following 5-day incubation at 20 °C. Calcium (Ca^2+^) and magnesium (Mg^2+^) levels were determined through complexometric titration, while Total Iron (Fe) was measured via the 1.10-phenanthroline spectrophotometric method.

Total iron (Fe) concentration was determined via UV–Vis spectrophotometry using the 1,10-phenanthroline method, as described in the Standard Methods for the Examination of Water and Wastewater (APHA, 2017). For concentrations exceeding 0.1 mg L^−1^, the precision of the method—expressed as Relative Standard Deviation (RSD)—was better than 5%. Accuracy, assessed through recovery tests, ranged between 95 and 105%. The method detection limit (MDL) was approximately 0.01 mg L^−1^ (APHA, 2017).

#### Carbonate system modeling and sensitivity indices

Dissolved CO₂ and bicarbonate (HCO₃⁻) concentrations were estimated using the CO2SYS software (v. 2.1; Lewis & Wallace, 1998), based on measured pH and total alkalinity (TA) as input parameters. Calculations were performed using the freshwater dissociation constants of Cai and Wang (1998). To address potential uncertainties under low pH conditions (pH < 5.3; Raymond et al., 2012), a conservative approach was adopted by excluding the upper 5th percentile of the CO₂ dataset to minimize non-linear error propagation (Orr et al., 2018). Results were further validated using the seacarb package to ensure thermodynamic consistency (Gattuso et al., 2021).

### Calcite saturation index (CSI)/ASI

The Calcite Saturation Index (CSI), hereafter referred to as the Acidification Sensitivity Index (ASI) when interpreted as a vulnerability metric, was employed to evaluate the intrinsic susceptibility of waters to acidification. The calculation followed the classical formulation presented by Clair et al. (1982) and later reviewed by Clair et al. (2007), which defines the equilibrium state of calcium carbonate in solution.

The index was calculated based on the negative logarithms of molar Ca^2+^ concentration (used as a proxy for activity) and total alkalinity (TA), adjusted for pH and an equilibrium constant (pK).

Although TA is expressed in equivalents per liter due to its definition as a charge balance parameter, Ca^2+^ was maintained in molar units, consistent with thermodynamic formulations of calcite saturation indices based on molar activities. In this formulation, TA is used as an operational proxy for carbonate alkalinity, representing the dominant buffering species (mainly HCO_3_^−^) in low-mineralized freshwater systems. While this approach does not explicitly resolve carbonate speciation, it provides a robust comparative index of buffering capacity and acidification sensitivity under field conditions, consistent with the framework proposed by Clair et al. (1982, 2007).

ASI values were interpreted according to standard thresholds, where values exceeding 3.0 indicate high sensitivity and values above 4.0 signify critical instability and chronic undersaturation.

The CSI was calculated using the following formula:

1 $$CSI=p\left[{Ca}^{2+}\right]+p\left[TA\right]-p\left[{H}^{+}\right]+pK$$

where: p[Ca^2+^]: negative logarithm of calcium concentration (− log [Ca^2^⁺], mol L^−1^);

p[TA]: negative logarithm of total alkalinity expressed in equivalents per liter (− log [TA]);

p[H^+^]: corresponds to the in situ pH;

*pK*: apparent equilibrium constant integrating calcite solubility and carbonate dissociation constants, adjusted for ionic strength and temperature (reference value ~ 2.0).

### Interpretation criteria

CSI < 1: Stable and well-buffered waters (saturated with respect to calcite).

CSI between 1 and 2: Waters with moderate sensitivity.

CSI 3 to 4: Waters highly sensitive to acidification.

CSI > 4: Critically sensitive and extremely unstable waters (state of strong undersaturation).

### Principal component analysis (PCA)

To diagnose the acidification status and identify the key physicochemical drivers governing lake variability, Principal Component Analysis (PCA) was applied. This multivariate statistical technique allowed for the dimensionality reduction of the 21 measured parameters, transforming them into new axes (components) that explain most of the data variance.

#### Procedure and mathematical framework

-Normalization: Due to the different measurement units (e.g., pH, Conductivity, Nutrients), the data were standardized using Z-score method to ensure all parameters had equal statistical weight (mean = 0, variance = 1).

2 $${Z}_{ij}= \frac{{X}_{ij}-{\overline{X} }_{j}}{{S}_{j}}$$

where ***Z***_***ij***_ is the standardized value, **x**_**ij**_ is the original measurement of parameter j, $\overline{{\boldsymbol{x}} }j$ the mean, and ***s***_***j***_ is the standard deviation.

-PCA Score Extraction (PC1): The first principal component (PC1) was selected as the "Status Index," as it captured the highest percentage of total variance. The score for each sample was calculated as a linear combination of the standardized variables:

3 $$PC1=\sum_{j=1}^{n}{w}_{j}{z}_{j}$$

where ***w***_***j***_ represents the loading (weight) of parameter ***j*** on the first component, and ***n*** is the number of included parameters. ***zj*** is the standardized value (Z-score) of the ***j***^***th***^ parameter for that sample, calculated previously in **Eq. **2. High positive loadings indicated the primary vectors of chemical alteration during periods of peak stress (e.g., dissolved CO_2_ and ionic load).

Amplitude calculation: To quantify the seasonal variability and chemical instability of each lake and layer, the Amplitude of the PC1 scores was calculated as follows:

4 $$Amplitudw PCI=PC1\left(max\right)-PC1\left(min\right)$$

This metric allowed for the identification of the most unstable compartments by comparing the annual oscillation range between the surface and bottom layers.

### Statistical analysis

Descriptive statistics, including mean, standard deviation, and coefficient of variation (CV), were calculated for all physicochemical parameters. To assess significant differences in water quality between the two lakes (AP vs. BL) and between sampling strata (surface vs. bottom), the non-parametric Mann–Whitney U test was applied. Statistical significance for all tests was defined at p < 0.05.

Principal Component Analysis (PCA) was performed to identify multivariate patterns and underlying biogeochemical processes. Prior to PCA, data were standardized (Z-score transformation) to account for the different units of the variables and to ensure they contributed equally to the model. The selection of significant principal components (PCs) was based on the Kaiser criterion (eigenvalues > 1). Factor loadings were used to interpret the influence of each variable on the extracted components. All statistical procedures were conducted using PAST® software (Version 4.0; Hammer et al., 2001).

## Results and discussion

### Precipitation patterns

**Fig. 2:**
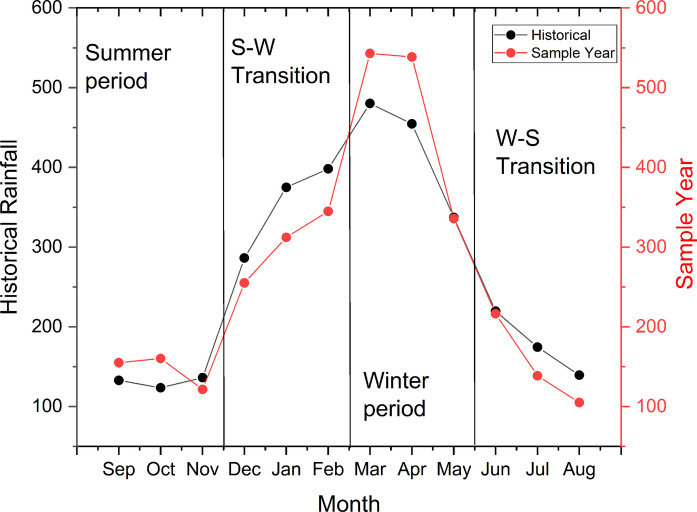
Temporal distribution of rainfall (mm) for the periods 2023–2024 (study period) and 1994–2024 (historical period).Data obtained from the INMET station near the study region (https://portal.inmet.gov.br/). The climatic periods were included for explanatory purposes in the results and later discussion. S: summer; W: winter

The hydrological regime during the 2023–2024 monitoring period was contextualized by comparing it against a 30-year historical dataset (1994–2024). Rainfall data for both periods were obtained from the INMET meteorological station located near the study area. Statistical evaluation using the Mann–Whitney U test (p = 0.84) indicated no significant deviation from long-term rainfall distributions. This parity confirms that the study period is representative of typical regional climatic cycles, ensuring that the observed biogeochemical dynamics reflect standard seasonal forcing rather than anomalous weather events (Fig. 2). Statistical analysis confirmed significant differences across the four climatic periods for both the historical rainfall series (H = 9.667, p = 0.0216) and the specific study period (H = 8.949, p = 0.0299). Post-hoc Dunn’s tests revealed that during the study period, the Winter period (March–May) differed significantly from the Summer (Sept–Nov; p = 0.0174) and the Winter-Summer Transition (June–August; p = 0.0127). For the historical data, the Winter period also showed distinct patterns compared to the Summer (p = 0.0046) and the Summer–Winter Transition (p = 0.0235). These findings reinforce that the intensification of rainfall during the winter months acts as a primary driver of environmental variability in the region, as illustrated in Fig. 2.

### Physicochemical characterization

**Fig. 3:**
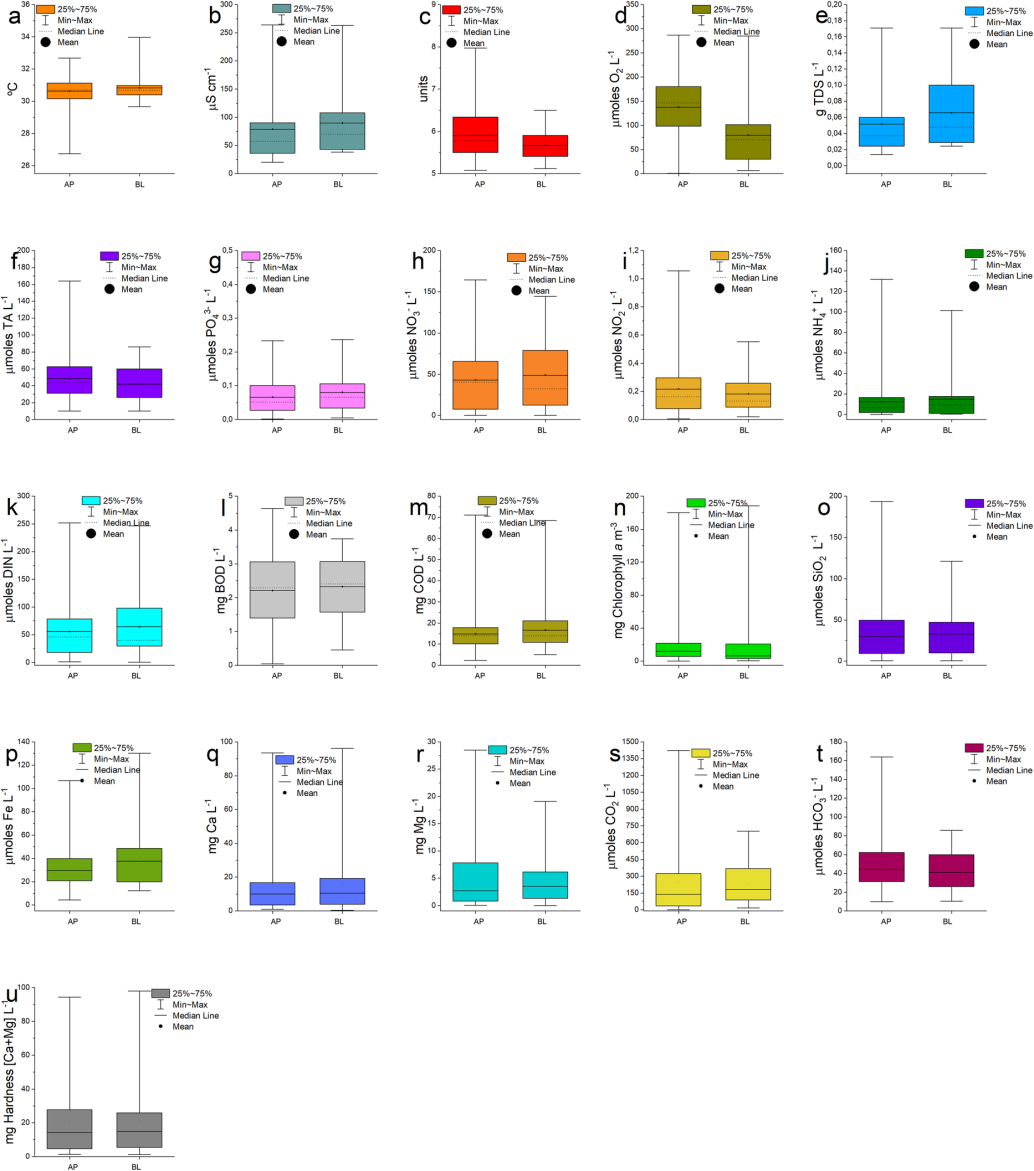
Distribution of water parameters in the Agua Preta and Bolonha lakes. The figure presents 21 physicochemical and biogeochemical variables monitored during the 2023–2024 period. Boxplots indicate Mean (indicated by the symbol and line), Median (horizontal line), Interquartile Range (25th–75th percentiles; box boundaries), and Minimum/Maximum values (whiskers). **a** Temperature (°C); **b** Electric conductivity; **c** pH; **d** Dissolved Oxygen-DO; **e** TDS; **f** Total Alkalinity-TA; **g** Phosphate-PO_4_^3−^; **h** Nitrate (NO_3_^−^); **i** Nitrite (NO_2_^−^); **j** Ammonium (NH_4_^+^); **k** DIN; **l** BOD; **m** COD; **n** Chlorophyll-*a*; **o** Silicate (SiO_2_); **p** Total Iron (Fe); **q** Calcium (Ca); **r** Magnesium (Mg); **s** Dissolved CO_2_ (CO_2_); **t** Bicarbonate (HCO_3_.^−^); **u** Hardness (Ca + Mg)

Water temperature across both systems ranged from 26.0 to 34.0 °C (mean: 30.6 ± 0.7 °C), with maximum values recorded during the dry season (September–November). Although both reservoirs exhibited similar mean temperatures (AP: 30.6 °C; BL: 30.8 °C), Lake Água Preta (AP) showed a larger thermal amplitude (~ 6.0 °C) compared to Lake Bolonha (BL; ~ 4.2 °C). However, no significant differences in temperature distributions were observed between lakes (Mann–Whitney U test, p = 0.41), consistent with Silva et al. (2022) (Fig. 3). The greater thermal variability in AP suggests enhanced temporal fluctuations in gas solubility and metabolic rates, indicating a system more responsive to atmospheric forcing.

Temperature variations influence reaction kinetics and gas solubility, particularly for CO_2_, which tends to decrease in solubility with increasing temperature. Thus, the wider thermal range observed in AP may contribute to greater variability in CO_2_ dynamics and associated pH fluctuations, especially during periods of intensified organic matter decomposition.

Regarding ionic composition, Lake Bolonha exhibited higher electrical conductivity (EC) and total dissolved solids (TDS), while Lake Água Preta showed lower absolute values but higher relative variability (Coefficient of Variation – CV = 75.8%) (Fig. 3; Table S1). This indicates that AP is more responsive to episodic inputs such as precipitation and runoff, reflecting lower ionic stability. In contrast, the higher EC and TDS values in BL suggest a more mineralized system, likely influenced by continuous external inputs from the watershed. Such variability is typical of low-mineralized systems with reduced buffering capacity, where chemical conditions are more sensitive to external forcing.

Consequently, temporal variability in EC and TDS was more pronounced in Lake AP (CV > 50%), reinforcing its higher sensitivity to environmental fluctuations and its stronger dependence on short-term climatic drivers. These parameters provide an important geochemical framework for interpreting pH dynamics and system resilience. As shown in Table S1, pH exhibited significant differences both between lakes (p = 0.0002) and between surface and bottom layers (p < 0.0001), highlighting the strong vertical and inter-system variability in acid–base conditions.

### Acidification-related processes and oxygen dynamics

The investigation revealed pronounced pH stratification across the water column, reflecting strong endogenous processes associated with CO_2_ production and oxygen consumption. pH levels fluctuated between 5.1 and 8.0 (mean: 5.9 ± 0.3), frequently failing to meet the CONAMA (2005) regulatory minimum of 6.0 for freshwater systems (Fig. 3). Highly significant differences were observed across all strata and between both lakes (p = 0.0002 for AP/BL; p < 0.0001 for surface/bottom layers). Notably, AP exhibited a greater vertical pH amplitude (0.40) than BL (0.27), suggesting more intense internal processes influencing hypolimnetic acidity.

This positive difference in both lakes indicates that pH values are generally higher at the surface and decrease with depth, although the magnitude of this gradient varies temporally. The greater vertical amplitude observed in AP suggests more pronounced vertical variability in acid–base conditions. This pattern is consistent with endogenous processes associated with dissolved oxygen (DO) depletion, as oxygen is consumed in bottom waters during organic matter decomposition, leading to CO_2_ production (Abril et al., 2014; Noriega et al., 2022).The resulting dissolved CO_2_ forms carbonic acid (H_2_CO_3_), acting as a primary acidifying agent in the hypolimnion. In both lakes, bottom waters were generally more acidic than surface waters, although this contrast is not uniform across all sampling periods.

This vertical gradient aligns directly with DO variation, which is also highly significant across the vertical profile (p < 0.05). The sharp decline in bottom DO indicates intense anaerobic decomposition of sedimented organic matter (Fig. S2; supplementary material).

Dissolved oxygen (DO) results further highlighted environmental stress (Table S1). Average DO levels in AP (138 ± 60 µmoles L^−1^) and BL (80 ± 46 µmoles L^−1^) were consistently below the legal threshold of 5.0 mg L^−1^ (~ 156 µmoles L^−1^). Approximately 62% of samples were non-compliant, with 5% exhibiting critical hypoxia (< 2.0 mg L^−1^), posing a substantial risk to aerobic biota. This chronic oxygen depletion facilitates the accumulation of dissolved CO₂, which hydrolyzes into carbonic acid, thereby lowering pH in bottom waters. The DO levels observed here are similar to those reported by Macedo et al. (2024) between 2011 and 2022 in lakes AP and BL.

BL exhibits a significantly lower average DO than AP, suggesting a more persistent oxygen deficit and greater susceptibility to hypoxic conditions. This difference is supported by the statistical analysis (p = 0.05 between lakes) and suggests that, while AP exhibits stronger vertical variability in acid–base conditions, BL may experience more persistent oxygen limitation, likely associated with higher organic loading and nutrient availability.

#### Carbon and nutrient cycling

Metabolic imbalances were confirmed by elevated dissolved CO₂ concentrations, averaging 217.5 µmoles L⁻^1^ in AP and 205.4 µmoles L⁻^1^ in BL (Fig. 3; Table S1; supplementary material). Maximum values were consistently detected at the bottom (AP_bottom_: 250.8 µmoles L⁻^1^), showing a strong inverse relationship with DO and establishing heterotrophic respiration as the primary driver of acidity. This is supported by high nutrient levels in the hypolimnion, where organic remineralization released NH₄⁺ (means: 2.3–2.6 µmoles L^−1^) and PO_4_^3−^ (means: 0.35–0.36 µmoles L^−1^) with high annual variability (CV: 65.48%–86.13%).

This demonstrates robust organic matter remineralization within the sediment and rapid nutrient cycling, characteristic of hypoxic/anoxic environments (Esteves, 2011; Melack & Forsberg, 2001; Noriega et al., 2022).

The release of NH_4_^+^ (which, depending on pH and temperature, may either buffer or contribute to acidity) and PO_4_^3−^ (released from Fe or Al complexes under acidic and reducing conditions) underscores the geochemical complexity of the bottom layer and validates the application of PCA to disentangle these interrelationships.

The lack of significant differences in Fe concentrations between the lakes (p = 0.33) indicates that both systems are influenced by a shared geogenic control. Nevertheless, Fe remains a critical factor in acidification processes due to its role in redox cycling. The relatively similar mean Fe concentrations (AP: ~ 43.2 µmoles L⁻^1^; BL: ~ 31.2 µmoles L⁻^1^) suggest that iron availability is not the primary driver of differences between the systems, but rather a common background condition modulating biogeochemical dynamics.

To contextualize these values, a conservative approach was adopted based on the environmental legal limit (CONAMA, 2005) for dissolved Fe (0.3 mg L⁻^1^, equivalent to 5.37 µmol L⁻^1^). Although the measured Total Fe concentrations are well below this threshold, they still represent a substantial pool of redox-active iron. Under hypoxic conditions, Fe is predominantly present in its reduced and soluble form (Fe^2+^), as the absence of oxygen inhibits the formation of insoluble ferric hydroxides such as Fe(OH)_3_ (Libes, 2009). This condition is particularly relevant in hypolimnetic waters, where low dissolved oxygen and reduced pH favor the persistence of dissolved Fe (Libes, 2009; Noriega et al., 2022).

During re-oxygenation events (e.g., seasonal turnover), the oxidation of Fe^2^⁺ releases protons and contributes to acidification, as described by the following reaction (**Eq. **5; Stumm & Morgan, 1996):

5 $${4Fe}^{2+}+{O}_{2}+{10H}_{2}O\to 4Fe{\left(OH\right)}_{3}+{8H}^{+}$$

This process represents a mechanism of secondary acidification, particularly relevant in systems with limited buffering capacity. Lake AP, which exhibits lower alkalinity (TA median: (~ 103.8 μmoles L^−1^) and greater pH variability (CV-pH ~ 6.50%), is therefore more susceptible to acidification driven by Fe redox transformations. In this context, the oxidation of Fe^2+^ can amplify the pH decline initiated by CO_2_ accumulation (Esteves, 2011).

Beyond the C–O–Fe interactions, the variability of dissolved nutrients further supports the presence of intense metabolic activity and internal loading processes. Ammonium (NH_4_^+^) and phosphate (PO_4_^3−^) exhibited high temporal variability (CV: 65.00–86.20% and 76.10–84.40%, respectively), with consistently elevated concentrations in bottom waters (Table S1). This pattern reflects pulsed nutrient release associated with organic matter remineralization under hypoxic conditions, linking eutrophication processes to acidification dynamics (Melack & Forsberg, 2001). In addition, nitrate (NO_3_^−^) in Lake AP showed considerable variability (CV ~ 54.30%), indicating instability in nitrogen cycling under low-alkalinity conditions.

The strong coupling between acid-generating processes (e.g., CO_2_ accumulation and Fe^2+^ oxidation) and nutrient dynamics (including potential PO_4_^3−^ release from Fe-bound phases under reducing conditions) highlights the complexity of these systems. As such, the acidification status cannot be adequately described using a single parameter.

To integrate the relative contributions of variables associated with acid–base conditions, mineralization, and nutrient enrichment, Principal Component Analysis (PCA) was applied to the full dataset. This multivariate approach enables the identification of dominant gradients controlling water chemistry and supports the development of a composite index to assess spatial and seasonal variability in water quality.

#### Principal component analysis (PCA): multivariate synthesis

The statistical utility of a Principal Component (PC) lies in its ability to consolidate the maximum degree of original variance onto a single axis, effectively transforming multi-dimensional data into a robust environmental index. For this study, the variance percentages explained by PC1 and PC2 for both reservoirs are detailed in Table S2. The justification for utilizing these components as indices (Table S2; Table 1; Table 2) was derived from a comprehensive PCA conducted on a standardized matrix of 14 parameters per lake. The resulting first components (PC1) were identified as statistically and ecologically significant proxies for quantifying acidification and general environmental variation.

**Table 1:** Factor loadings of the first two principal components (PC1 and PC2) for the physicochemical parameters in Lake Água Preta. Values in **bold** denote parameters with the most significant weight in defining the multivariate structure

Parameter	PC1​	PC2​
Dissolved CO_2_	**0.457**	0.205
pH	**−0.448**	−0.045
Temperature	**−0.387**	0.082
Dissolved Oxygen-DO	**−0.369**	−0.004
NO_3_^−^	**0.330**	−0.255
Fe	**0.261**	0.025
TA	0.220	**0.429**
NH_4_^+^	**0.161**	0.020
Mg^2+^	−0.181	**0.343**
SiO_2_	0.086	**0.280**
EC	−0.087	**0.386**
Ca^2+^	0.067	**−0.250**
PO_4_^3−^	−0.022	**0.400**
NO_2_^−^	−0.019	**0.363**

**Table 2:** Factor loadings of the first two principal components (PC1 and PC2) for the physicochemical parameters in Lake Bolonha. Values in **bold** denote parameters with the most significant weight in defining the multivariate structure

Parameter	PC1​	PC2​
NO_2_^−^	**0.435**	−0.063
EC	**0.429**	−0.057
PO_4_^3−^	**0.402**	−0.233
NO_3_^−^	**−0.392**	0.038
Fe	−0.272	**−0.401**
Temperature	**0.247**	0.149
Mg^2+^	**0.208**	−0.061
pH	0.186	**0.217**
NH_4_^+^	−0.183	**0.193**
Ca^2+^	**−0.183**	0.158
Dissolved CO_2_	−0.144	**−0.460**
SiO_2_	0.088	**−0.244**
Dissolved Oxygen-DO	0.049	**0.390**
TA	0.028	**−0.467**

#### Benchmarks for significance

The relevance of each PC was rigorously evaluated against two established benchmarks:

*Explained variance criterion*: According to Abdi and Williams (2010) and Hair et al. (2019), in a complex environmental dataset with 14 variables, a single PC is considered robust if it explains significantly more than the mean variance per variable (Average variance per variable: 100%/14 variables ≈ 7.14%).

The observed results showed that PC1 for AP (21.05%) and PC1 for BL (23.87%) explained three times more variance than what would be expected from a random variable (~ 7.14%). Both PC1s (as well as PC2 for BL) concentrate the majority of the system's ‘signal,’ validating their use as a synthetic index.

*The Kaiser Criterion* (Eigenvalue > 1; Wang et al., 2025): the Kaiser Criterion states that a PC is significant if its eigenvalue (the variance it carries) is greater than 1. Since the explained variance (%) is the eigenvalue divided by the total variance, validity can be inferred as follows:

Eigenvalue = [Explained variance (%)/100] × Number of variables.

Consequently, AP (PC1) = [21.05/100] × 14 = 2.95, and BL (PC1) = [23.87/100] × 14 = 3.34. Both eigenvalues (2.95 and 3.34) are significantly greater than 1, confirming that the PC1s represent a real underlying factor and are statistically valid.

The PCA performed on the full dataset (including both surface and bottom samples) was used to identify the dominant gradients structuring the system as a whole, particularly gradients associated with vertical differentiation. In this context, PC1 explained 21.05% of the total variance and represents the primary axis of system-wide variability. (Table S2; Table 1). PC1 can be interpreted as being associated with stratification and CO_2_-related acidification processes, exhibiting positive loadings for dissolved CO_2_ (~ 0.457) and NO_3_^−^ (~ 0.330), and negative loadings for pH (~ −0.448), temperature (~ −0.387), and dissolved oxygen (DO; ~ −0.369).

These relationships suggest that variability captured by PC1 is linked to vertical gradients in the water column. Higher PC1 scores tend to be associated with conditions commonly observed in deeper waters, where oxygen consumption is coupled with CO₂ accumulation and lower pH. This pattern is consistent with metabolic processes related to organic matter decomposition and supports the interpretation of PC1 as an index associated with acidification dynamics in Lake AP.

In BL, PC1 accounted for 23.87% of the total variance (Table S2; Table 2), and is characterized by strong positive loadings for NO_2_^−^ (0.435), electrical conductivity (EC ≈ 0.429), and PO_4_^3−^ (0.402), while the negative axis exhibits inverse correlations with NO_3_^−^ (−0.392) and Total Fe (−0.272). This multivariate structure suggests that, in contrast to Lake AP, pH is not the dominant factor structuring PC1 in the Bolonha system. Instead, PC1 can be interpreted as a gradient associated with eutrophication–mineralization processes, reflected by the covariation of nutrients and dissolved ions (EC, PO_4_^3−^, NO_2_^−^), in opposition to NO_3_^−^ depletion and concurrent Fe variability under reducing conditions.

Geochemical acidification and redox dynamics are more prominently represented in PC2 (20.43%), which shows strong negative loadings for dissolved CO_2_, total alkalinity (TA), and Fe, alongside a positive correlation with dissolved oxygen (DO). This pattern is consistent with a redox gradient linking oxygenated conditions (positive DO) to bottom-water environments associated with enhanced respiration, CO₂ accumulation, reduced buffering capacity, and increased Fe solubility.

Collectively, these results suggest that PC1 defines the primary axis of variability in BL, associated with eutrophication–mineralization processes (23.87%), whereas PC2 captures secondary variability related to redox-driven acidification and buffering dynamics (20.43%). Along this axis, Fe covaries with CO_2_ and TA, responding to shifts between oxic and reducing conditions rather than acting as an independent driver of acidification.

#### Principal components analysis by a water layer

Four discrete PCA models were constructed using the comprehensive 14-parameter matrix to identify dominant patterns of chemical variability within specific environmental compartments. In each model, the first principal component (PC1) can be interpreted as a layer-specific index of physicochemical variability, often associated with acidification-related and broader biogeochemical processes.

For Lake Agua Preta (AP), surface-layer results indicated that PC1 accounted for 20.44% of the total variance, reflecting variability linked to carbon dynamics and buffering conditions. Dissolved CO_2_, TA, Fe, and SiO_2_ exhibited the strongest positive loadings, whereas pH and dissolved oxygen (DO) were associated with the negative axis (Table S3). This structure suggests an inverse relationship between CO_2_ and pH, consistent with acidification-related variability under limited buffering conditions.

In the bottom layer, PC1 explained 23.60% of the variance and is consistent with variability associated with metabolic processes and oxygen gradients. Here, pH, DO, and temperature displayed the highest positive loadings, while CO₂ and NO₃⁻ were negatively associated with the axis. This pattern is consistent with increased respiration and CO₂ accumulation under low-oxygen conditions.

The main parameters contributing to PC1 variability in Lake AP are summarized in Table 3.

**Table 3:** Summary of PC1 loadings and interpretation for the surface and bottom layers in Lake Agua Preta

Parameter	AP Surface (PC1​)	AP Bottom (PC1​)	Interpretation
Dissolved CO_2_	0.508	−0.332	Strong contributor to PC1 variability; associated with carbon dynamics across layers
pH	−0.442	0.426	Inversely related to CO_2_, defining the acidification gradient
TA	0.446	0.006	Closely associated with CO_2_ at the surface; weak contribution at depth
DO	−0.269	0.382	Contributes to PC1 variability, particularly in bottom waters, reflecting oxygen gradients
NO_3_^−^	0.183	−0.351	Moderate contribution; indicates variability in nitrogen dynamics, especially at depth
Fe	0.266	−0.223	Secondary contributor; reflects redox-related variability rather than a dominant control
Variance PC1	20.44%	23.60%	-

In Lake Bolonha (BL), surface-layer PC1 is largely associated with variability in dissolved ions and nutrients, consistent with the higher ionic variability and alkalinity observed in the system (Table S2; Table S5; Supplementary Material). Strong positive loadings were observed for NO_2_^−^ and EC, while Fe, NO_3_^−^, and CO_2_ were negatively associated with the axis (Table S5).

In the hypolimnion of BL (PC1: 26.28% variance), the multivariate structure reflects variability linked to nutrient and ionic dynamics. While NO_2_^−^, PO_4_^3−^, EC, and TA showed strong negative loadings, NO_3_^−^⁻ and NH_4_^+^ were associated with the positive axis (Table 4). This pattern suggests a gradient of nutrient transformation and ionic variability rather than a direct pH-driven structure.

**Table 4:** Summary of PC1 loadings and interpretation for the surface and bottom layers in Bolonha Lake (BL)

Parameter	BL Surface (PC1​)	BL Bottom (PC1​)	Interpretation
EC	0.408	−0.352	Strong contributor to PC1 variability; reflects changes in dissolved ionic content
NO_2_^−^	0.411	−0.415	Major contributor; associated with variability in nitrogen cycling
PO_4_^3−^	0.310	−0.396	Contributes to PC1 variability; associated with nutrient dynamics
TA	−0.208	−0.346	Moderate contribution; reflects variability in buffering conditions, particularly at depth
Dissolved CO_2_	−0.339	−0.258	Contributes to variability; secondary relative to nutrient and ionic parameters
Fe	−0.325	0.056	Secondary contributor; reflects redox-related variability, with limited influence on PC1
Variance PC1	25.79%	26.28%	-

While these analyses highlight the dominant spatial and multivariate structure of the systems, temporal variability associated with the regional rainfall regime represents an additional key driver of lake dynamics and is examined in the following section.

The hypolimnetic structure of Lake Bolonha (BL) differs from that of Lake Água Preta (AP), as supported by both univariate spatial comparisons (Table S1) and multivariate PCA results. Table S1 indicates that BL is characterized by higher ionic strength and mineralization (e.g., higher EC, TDS, and alkalinity), whereas AP exhibits lower buffering capacity and greater relative variability. These spatial differences provide the geochemical context for the distinct multivariate structures observed in each system.

In AP, the primary structure is associated with the CO_2_–pH relationship, reflecting the dominance of carbon-driven metabolic processes in a low-alkalinity environment. In contrast, PC1 in BL represents a gradient of eutrophication–mineralization, characterized by the covariation of nutrient concentrations (NO_2_^−^, PO_4_^3−^) and dissolved ions (EC). This indicates that, in BL, variability is more strongly controlled by nutrient inputs and ionic composition than by carbon dynamics alone.

These results suggest that the higher mineralization and nutrient availability in BL, as evidenced in Table S1, shift the dominant control from carbon-driven acidification (AP) to nutrient–ionic dynamics (BL). High loadings for NO_2_^−^ and PO_4_^3−^ are consistent with active organic matter remineralization and dynamic nitrogen cycling in bottom waters. Although dissolved CO₂ is also associated with this axis, PCA results indicate that nutrient and ionic variability represent the dominant structure in PC1, while carbon-related processes are more clearly expressed in secondary components (e.g., PC2; Table S6; Supplementary Material).

#### Integrative synthesis: PCA scores and the acidification sensitivity index (ASI)

The combined application of Principal Component Analysis (PCA), representing ecosystem state, and the Acidification Sensitivity Index (ASI), representing chemical vulnerability, provides an integrated framework for understanding acidification dynamics in Lakes AP and BL, extending beyond the descriptive capacity of isolated pH measurements. This dual-index approach enables the distinction between stress intensity (captured by PCA) and structural fragility (represented by ASI), offering a more comprehensive diagnostic perspective than univariate analyses.

PCA results suggest that, although both lakes undergo acidification processes, the associated mechanisms differ between systems (metabolic acidification vs. nutrient-driven variability).

In Lake AP, multivariate patterns indicate that variability in acid–base conditions is largely associated with the inorganic carbon cycle. Heterotrophic decomposition of organic matter, particularly within the hypolimnion, contributes to CO_2_ accumulation, which is associated with pH reduction under limited buffering conditions. This metabolic behavior is consistent with observations from other Amazonian blackwater and clearwater systems, where respiration may exceed primary production, contributing to CO_2_ enrichment (Melack & Forsberg, 2001).

In Lake BL, PC1 is primarily associated with nutrient and ionic variability (NO_2_^−^, PO_4_^3−^, and EC), suggesting that acidification processes are linked to broader water quality conditions. Elevated nutrient availability can enhance surface primary production, occasionally increasing pH, while also promoting organic matter accumulation and subsequent decomposition in bottom waters. This process is associated with oxygen consumption and CO_2_ production, contributing to hypoxic and lower-pH conditions in the hypolimnion. Such interactions between nutrient enrichment, metabolic processes, and buffering capacity are commonly reported in urban tropical reservoirs (Marotta et al., 2012; Paranaíba et al., 2021).

ASI results indicate that both reservoirs operate under conditions of chronic chemical vulnerability (ASI < 0), associated with relatively low alkalinity in relation to Ca^2^⁺ concentrations (Table 5). The high amplitude of PCA scores in Lake AP, combined with low ASI values, suggests increased sensitivity to acidification during precipitation-driven dilution events. In contrast, although BL often exhibits higher mean pH values, lower ASI values in bottom waters (e.g., ASI ≈ −1.05; Table 5) indicate reduced buffering capacity. This pattern may reflect a condition of apparent stability, in which biological CO_2_ uptake partially offsets acidity at the surface, while the system remains vulnerable to disturbances such as intense rainfall or mixing events.

**Table 5:** Comparative summary of PCA scores and ASI vulnerability indices for Lakes Água Preta (AP) and Bolonha (BL)

Compartment	PCA range	PCA amplitude	ASI range	ASI amplitude	ASI mean
Água Preta	−4.286; 5.667	9.953	−2.456; 0.308	2.764	−1.028
Bolonha	−4.710; 3.006	7.716	−2.567; −0.174	2.393	−1.095
Bottom [AP + BL]	−4.710; 4.885	9.595	−2.567; 0.159	2.726	-
Surface [AP + BL]	−3.471; 5.667	9.137	−2.456; 0.209	2.665	-
Bottom [AP]	−4.130; 5.465	9.595	−2.566; 0.159	2.726	−1.022
Surface [AP]	−2.815; 5.823	8.638	−2.605; 0.060	2.665	−1.035
Bottom [BL]	−3.511; 4.187	7.699	−2.466; −0.105	2.360	−1.047
Surface [BL]	−3.314; 4.205	7.519	−2.488; −0.095	2.393	−1.043

#### Regional and global contextualization

The biogeochemical patterns observed in these reservoirs align with broader tropical limnology research. The CO_2_-driven acidification observed in AP reflects findings by Abril et al. (2014) in Amazonian floodplains, where sediment respiration and flooding extent modulate carbon dynamics. Furthermore, the decoupling of oxygen and carbon cycles in BL mirrors results from eutrophic Brazilian reservoirs, where the hypolimnion functions as an acidic ‘anaerobic reactor’ (Almeida et al., 2019). The metabolism of Lake AP also finds parallels in stratified African systems, such as the Congo Basin, where heterotrophic respiration is the primary driver of pH suppression (Borges et al., 2015). This reinforces the importance of monitoring these parameters as indicators of environmental change, consistent with multivariate patterns identified in Lake AP.

#### Implications for watershed management

The combined use of PCA and ASI highlights limitations of traditional monitoring approaches based solely on isolated parameters, suggesting the need for more integrative strategies in managing these metropolitan systems. Based on the observed patterns, a differentiated management approach can be considered:

Lake AP: Watershed conservation and erosion control may help reduce the influx of allochthonous organic matter, with dissolved CO_2_ serving as a potential early warning indicator of metabolic imbalance.

Lake BL: Management efforts should prioritize the reduction of external nutrient inputs, particularly through improvements in sanitation infrastructure, to mitigate eutrophication processes associated with hypolimnetic CO₂ accumulation and reduced buffering capacity.

Understanding how these geochemical and metabolic mechanisms vary under the influence of the Amazonian hydrological cycle is essential. The following section examines the temporal evolution of these parameters, with particular attention to periods of increased variability, such as the transition between dry and wet seasons.

#### Temporal variability

**Fig. 4:**
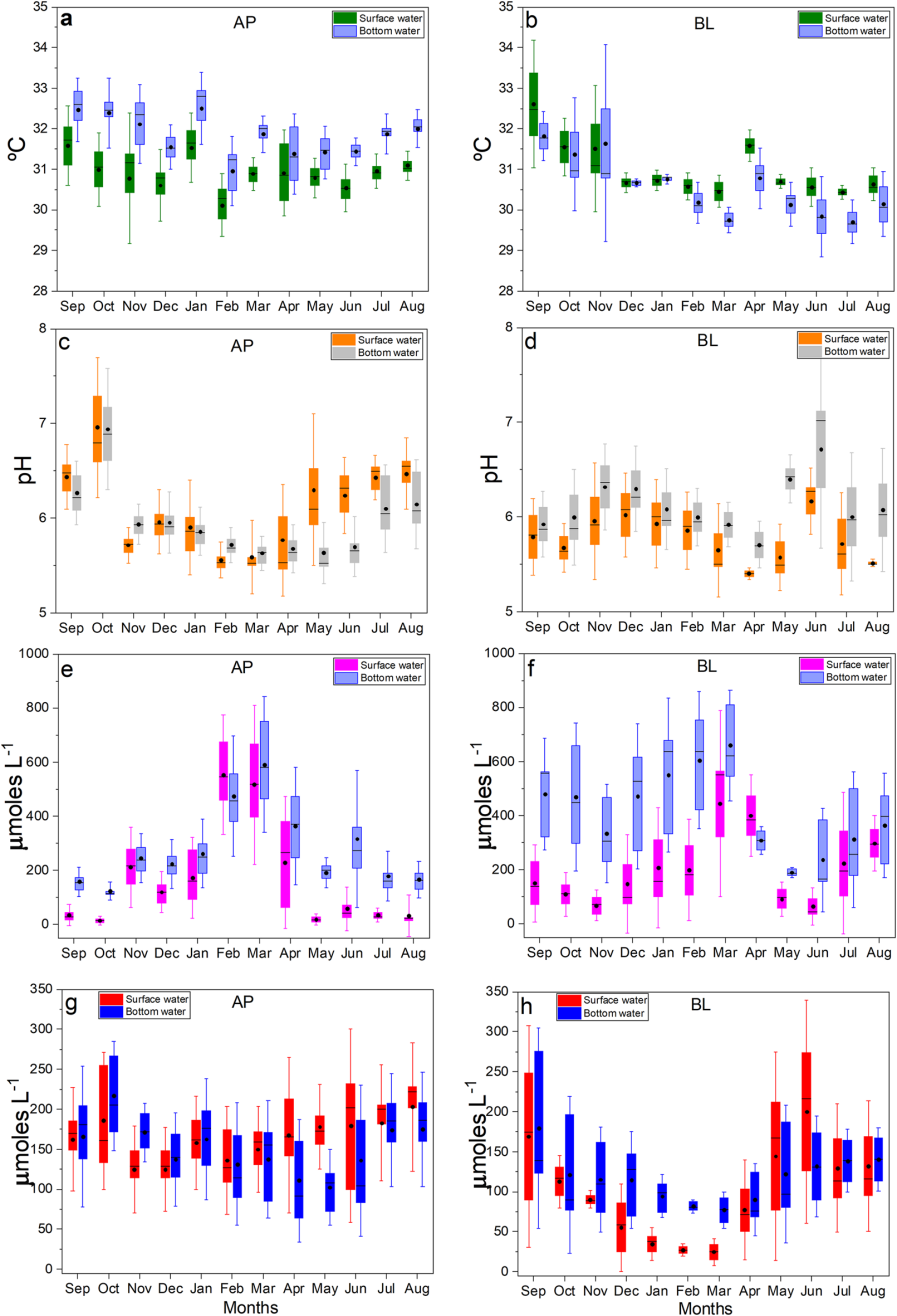
Monthly distribution of physicochemical parameters in surface and bottom waters of Lakes Água Preta (AP) and Bolonha (BL). Panels **a–b** show temperature (°C), **c–d** pH, **e–f** dissolved CO_2_ (µmol L^−1^), and **g–h** dissolved oxygen (µmol L^−1^). Boxplots represent the median (central line), interquartile range (box), and variability (whiskers, 1.5 × IQR), with individual points indicating observed values. Black circles denote mean values. The plots illustrate seasonal variability and highlight differences in temporal behavior between lakes and water layers, as well as the degree of overlap between surface and bottom conditions

The two lakes exhibit distinct temporal behaviors (Fig. 4), reflecting differences in their geochemical structure and dominant controlling processes. Lake Água Preta (AP), characterized by lower alkalinity and higher relative variability (Table S1), shows temporal dynamics strongly driven by metabolic processes, particularly carbon cycling (CO_2_–pH–DO). In contrast, Lake Bolonha (BL), with higher ionic strength and nutrient concentrations, exhibits variability more closely associated with mineralization and eutrophication processes.

Detailed temporal analysis elucidates how the hydrological cycle modulates environmental risks, identifying two distinct critical periods driven by opposing biogeochemical parameters. Time-series data reveal a strong coupling between the regional rainfall regime and the chemical stability of the lakes. During the dry season (September–November; Fig. 2), the reduction in precipitation coincides with peak PCA scores (maximum positive values). This escalation was primarily driven by elevated dissolved CO_2_ and a concomitant decline in DO, particularly within Lake AP (Figs. 4e, f, g, h). Lower water volumes and higher temperatures accelerate metabolic rates, thereby concentrating on the effects of organic decomposition (Fig. S1; Figs. 4a, 4b). Consistent with observations by Abril et al. (2014) in other Amazonian aquatic systems, ecosystem respiration acts as a primary driver of carbon emission and acidification. This process is further exacerbated in tropical systems by the significant load of allochthonous organic matter leached from the surrounding forest. Additionally, the proximity of urban settlements with inadequate sanitation infrastructure must be considered an intensifying factor (Fig. 1; ANA, 2017).

**Fig. 5:**
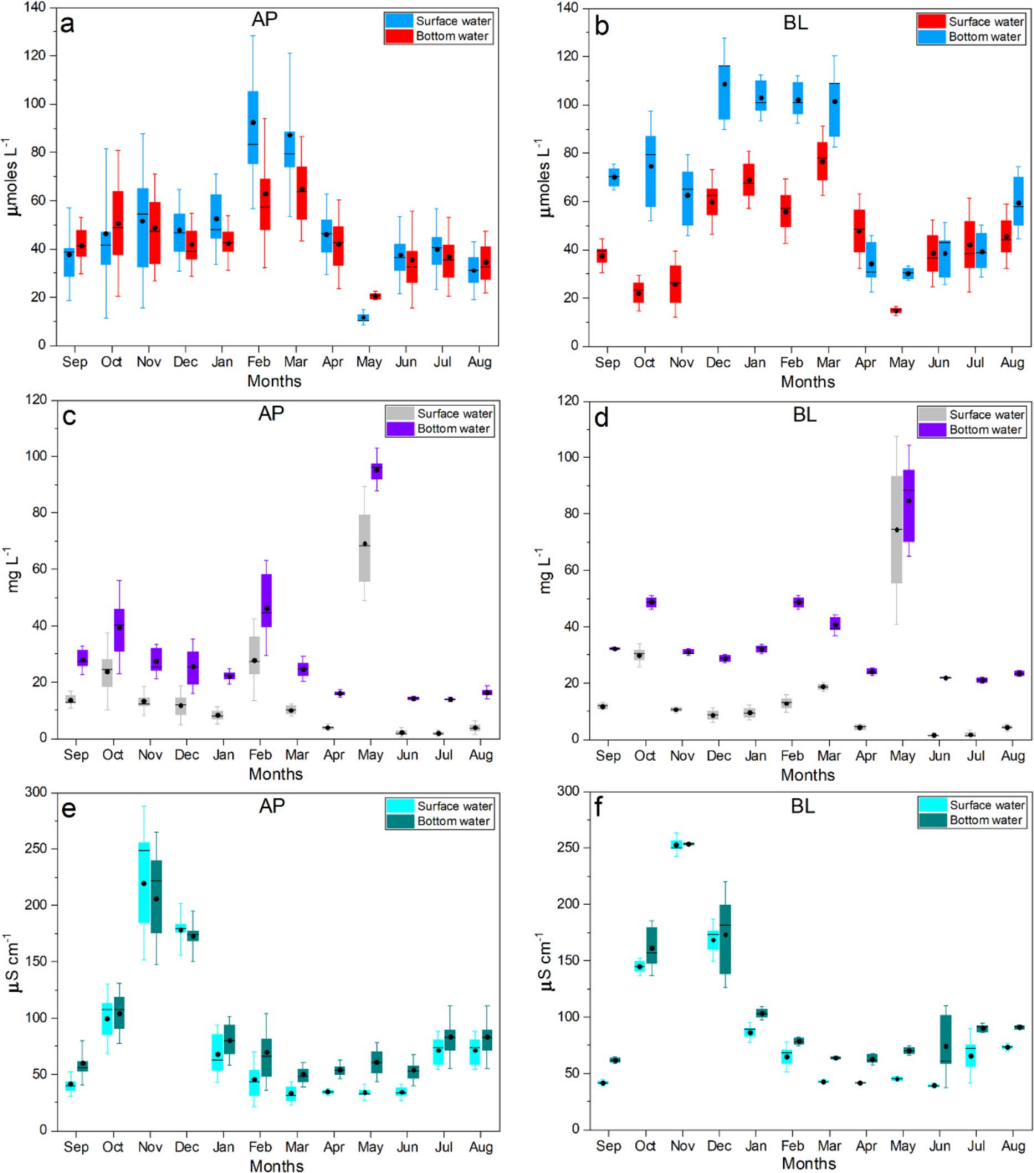
Monthly distribution of parameters related to ionic composition and buffering capacity in surface and bottom waters of Lakes Água Preta (AP) and Bolonha (BL). Panels **a–b** show Total Alkalinity (TA; µmol L^−1^), **c–d** Ca^2+^ (mg L^−1^), and **e–f** electrical conductivity (EC; µS cm^−1^). Boxplots represent the median (central line), interquartile range (box), and variability (whiskers, 1.5 × IQR), with individual points indicating observed values. Black circles denote mean values. The plots highlight seasonal variability, differences in dilution response between lakes, and the degree of overlap between surface and bottom layers

**Fig. 6:**
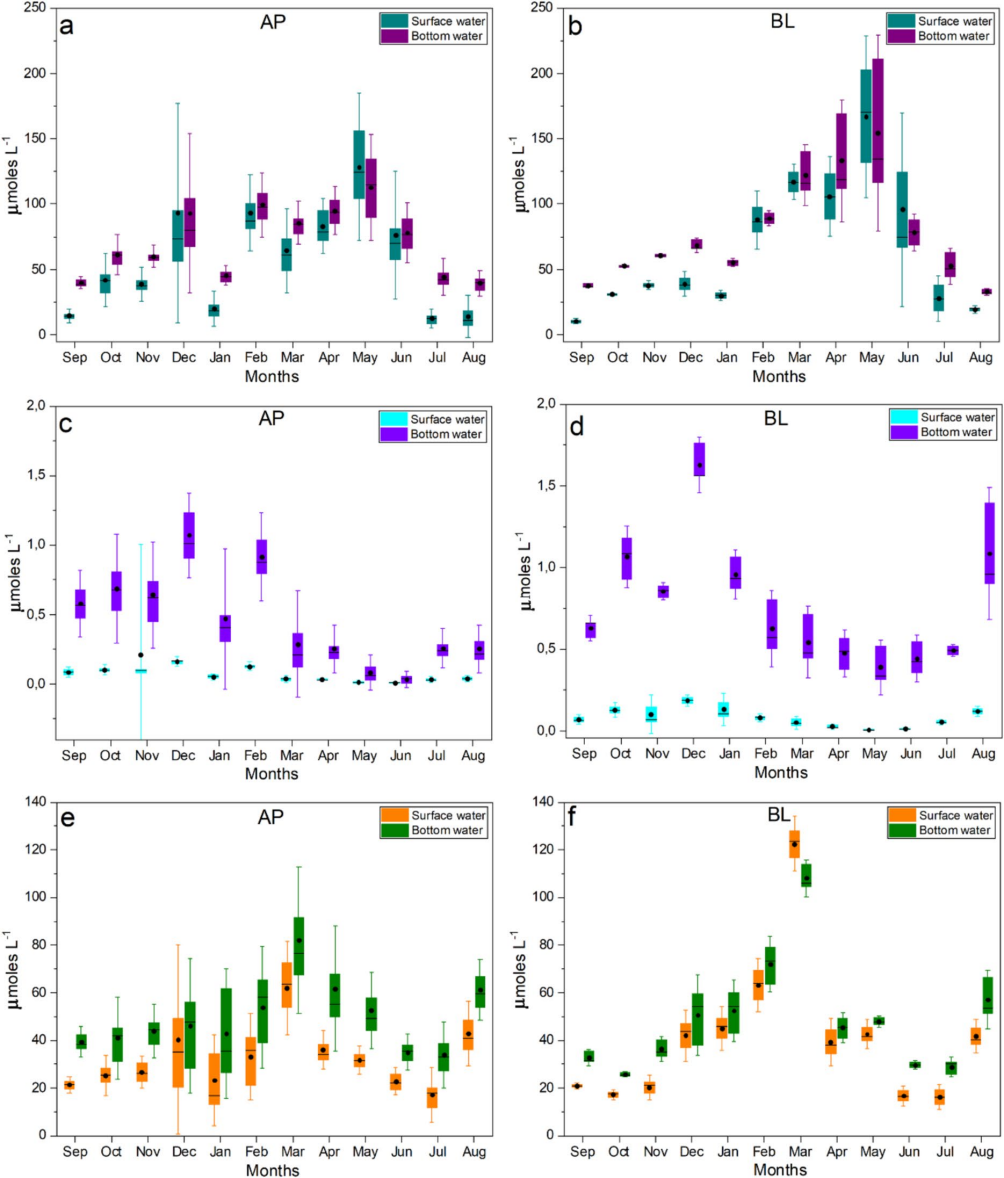
Monthly distribution of nutrients and iron (Fe) in surface and bottom waters of Lakes Água Preta (AP) and Bolonha (BL). Panels represent dissolved inorganic nitrogen (DIN), phosphate (PO_4_^3−^), and total iron (Fe) concentrations (units as indicated). Boxplots represent the median (central line), interquartile range (box), and variability (whiskers, 1.5 × IQR), with individual points indicating observed values. Black circles denote mean values. The plots illustrate temporal variability associated with hydrological forcing and highlight differences in nutrient dynamics and metal mobilization between lakes and water layers

Vertical comparisons suggest the occurrence of thermal and chemical stratification, with surface waters generally associated with higher dissolved oxygen and pH, and bottom waters reflecting conditions linked to organic matter decomposition (Fig. 4). However, these surface–bottom differences vary over time and are not consistently maintained across all months. In the surface layer, higher DO and pH values are associated with atmospheric exchange and photosynthetic CO_2_ uptake (Figs. 4c, d, g, h). In contrast, bottom waters tend to exhibit higher concentrations of dissolved CO_2_, electrical conductivity (EC), and nutrients such as NH_4_^+^ and PO_4_^3−^, reflecting mineralization processes (Figs. 5e–f; Fig. 6c–d; Fig. S2). As highlighted by Esteves (2011), the relative isolation of the bottom layer can limit re-oxygenation, favoring reducing conditions and the accumulation of CO₂ and other products of organic matter degradation, which are associated with lower pH values.

During the wet season (March–May), the Acidification Sensitivity Index (ASI) tends to reach its lowest values, indicating increased susceptibility to acidification. However, the magnitude and expression of this response differ between the lakes (Figs. 5a–d), reflecting contrasts in their baseline geochemical characteristics. Lake Água Preta (AP), characterized by lower alkalinity and reduced ionic strength, exhibits a more pronounced dilution effect, where the influx of meteoric water rapidly decreases total alkalinity (TA) and Ca^2+^, leading to a sharp decline in buffering capacity. In contrast, Lake Bolonha (BL), with higher mineralization and ionic content, shows a comparatively attenuated response, although reductions in TA are still observed, particularly in bottom waters. The influx of meteoric water promotes dilution of total alkalinity (TA) and Ca^2+^, contributing to reduced buffering capacity, a process widely described for low-mineralized systems (Stumm & Morgan, 1996). This dilution of basic cations reduces the Acid Neutralizing Capacity (ANC), thereby increasing the system’s sensitivity to pH fluctuations. Figure 5 shows the main parameters related to ionic stability, indicating that bottom waters are often associated with lower buffering conditions, although this pattern varies temporally. While electrical conductivity (EC) may be slightly higher at depth due to ion accumulation from sediment processes, reductions in TA are also observed in the hypolimnion (e.g., in Lake Bolonha, from ~ 45 to ~ 39 µmol L^−1^), suggesting bicarbonate (HCO_3_^−^) consumption during organic matter degradation. The combination of relatively low Ca^2+^ concentrations (< 16 µmol L^−1^) and reduced alkalinity reflect conditions of increased ionic vulnerability, in which the buffering capacity is diminished, and the system becomes more sensitive to proton inputs associated with CO_2_ production.

The CO_2_ and DO dynamics suggest that acidification in Lake AP is strongly associated with metabolic processes (Figs. 4e–h). During the dry-to-wet transition (December–February), the onset of rainfall increases the input of terrestrial organic matter into the reservoir, helping maintain relatively elevated CO_2_ concentrations despite increased water volume. Time-series analysis of nutrients (DIN and PO_4_^3−^) and Fe indicates a clear relationship between the hydrological regime and material fluxes (Fig. 6). DIN concentrations tend to increase during the wet season (March–May), coinciding with periods of higher precipitation, suggesting that nitrogen inputs are influenced by surface runoff from both urban and forested areas. As noted by Yang et al. (2020) and Esteves (2011), rainfall acts as an important transport vector for nutrients from the watershed into aquatic systems, contributing to increased NO_3_^−^ and NH_4_^+^ concentrations (Figs. S3–S4; Supplementary Material).

In contrast, PO43- concentrations tend to peak during the late dry season and transition phase (November–December). This pattern may be associated with evaporative concentration and internal sediment loading under reduced water volume conditions, a process commonly reported in tropical reservoirs (Callisto et al., 2014; Tundisi & Tundisi, 2008). Iron (Fe) exhibits a pulse-like temporal pattern, with higher concentrations observed at the onset of the wet season (e.g., March), consistent with increased leaching of lateritic soils following initial rainfall events (Junk et al., 2011). Although Fe concentrations do not differ significantly between lakes, its temporal variability appears to reflect both changes in redox conditions and external inputs. Its solubility is enhanced under acidic and reducing conditions, particularly in bottom waters. As discussed by Abril et al. (2014), the combined influx of metals and organic matter during this transition can alter redox balance and ionic conditions, contributing to variability in buffering capacity reflected in the ASI.

Vertical profiles indicate that the hypolimnion acts as an important zone for the accumulation and transformation of nutrients and metals, as commonly observed in stratified tropical lakes under limited mixing conditions (Esteves, 2011). While surface concentrations may be partially reduced by biological uptake, bottom waters are often associated with relatively higher concentrations during stratified periods, although this pattern varies temporally. For Fe, elevated bottom-water concentrations (e.g., up to 42.8 µmol L^−1^ in Lake AP) are consistent with sediment remobilization under low-oxygen conditions (< 5 mg L^−1^), as described by Esteves (2011). This process contributes to vertical chemical gradients and, together with CO_2_ accumulation, is associated with increased chemical instability in deeper layers.

In Lake Bolonha, where PC1 is primarily associated with nutrient dynamics, the observed temporal patterns are consistent with stronger urban influence. Tundisi and Tundisi (2008) describe how urban reservoirs in Brazil are affected by cultural eutrophication, in which nitrogen and phosphorus inputs alter oxygen and carbon dynamics, often promoting hypoxic bottom conditions. In this system, nutrient variability (e.g., NO_2_^−^ and PO_4_^3−^) appears to be linked to periods of reduced dilution and potential urban inputs. In tropical urban reservoirs, reduced water volume during drier periods may intensify nutrient concentration effects, contributing to eutrophication and associated changes in buffering capacity (Callisto et al., 2014; Tundisi & Tundisi, 2008).

**Fig. 7:**
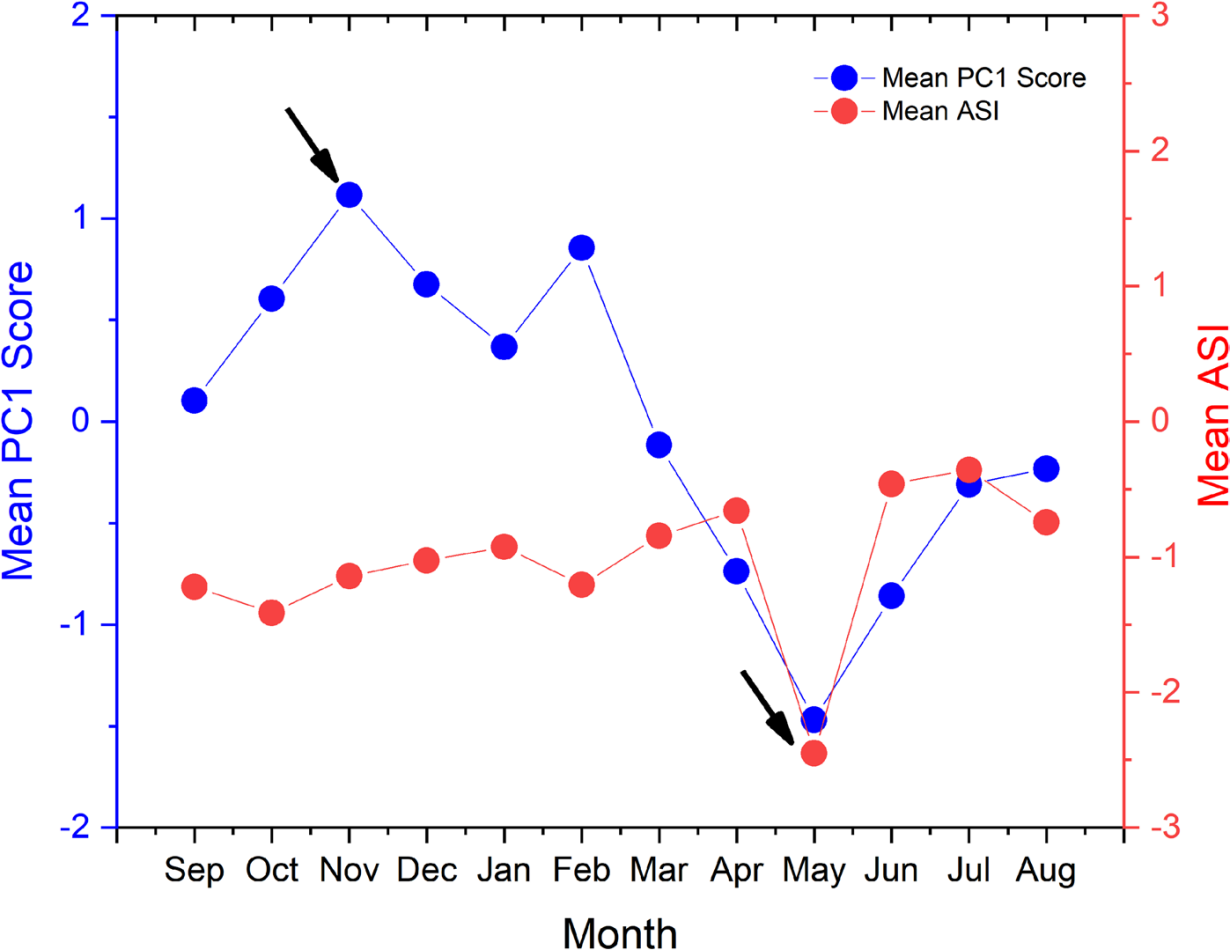
Time series of PCA scores and ASI indices estimated for the AP and BL systems during 2023–2024 (monthly mean scores). Arrows denote the extreme values for these indices

The ASI profile shows an inverse relationship with precipitation patterns (Figs. 2, 3, 4, 5, 6 and, 7), with lower ASI values generally associated with periods of higher rainfall. This pattern suggests that rainwater, typically characterized by low electrolyte concentrations, contributes to the dilution of the lakes’ buffering capacity. In tropical systems, this vulnerability may not be fully reflected in pH values alone (Abril et al., 2014; Esteves, 2011). However, analysis of the alkaline reserve indicates that periods of intense precipitation can reduce the Acid Neutralizing Capacity (ANC), increasing the system’s sensitivity to pH fluctuations. Overall, the time series for Lakes AP and BL suggests a seasonal contrast (Scissor effect): during the dry season, conditions are strongly associated with biogeochemical processes (e.g., higher CO_2_ and lower DO), whereas during the wet season, reduced alkalinity and lower ASI values indicate increased geochemical vulnerability (Fig. 7).

Integrated time-series analysis suggests that Lakes Água Preta and Bolonha operate under a regime of dual seasonal criticality, governed by the alternation between metabolic stress and ionic fragility. This pattern, observed across Figs. 4, 5, 6, and 7, appears to be influenced by the regional rainfall regime. During low-precipitation months, reduced water volume and higher temperatures are associated with increased heterotrophic activity. Patterns observed in Fig. 4 (higher CO_2_ and lower DO) and Fig. 6 (increased nutrients and Fe variability) coincide with elevated PCA scores (Fig. 7), indicating periods of intensified biogeochemical activity. During this phase, acidification is largely associated with metabolic processes. As described by Esteves (2011), thermal stability during the dry season can limit vertical mixing, favoring oxygen depletion and the accumulation of CO_2_ in bottom waters.

With the onset of intense rainfall, a contrasting pattern is observed. Increased water input promotes dilution of nutrients and Fe, while also reducing concentrations of basic cations and total alkalinity (TA) (Fig. 5). As a result, ASI values tend to decrease (Fig. 7), indicating greater sensitivity to acidification. Although PCA-derived stress indicators may decline due to increased mixing and re-oxygenation, the system exhibits reduced buffering capacity. According to Stumm and Morgan (1996), dilution of Ca^2+^ and HCO_3_^−^ can weaken buffering conditions, increasing susceptibility to pH fluctuations. Vertical comparisons (Figs. 4, 5, 6) indicate that bottom waters are generally associated with conditions of lower dissolved oxygen and reduced buffering capacity, although surface–bottom differences vary over time. Figure 7 further supports this pattern, showing greater variability in the amplitude of indices at the bottom compared to the surface.

While Lake AP is primarily influenced by carbon cycling and metabolic processes (Abril et al., 2014), Lake Bolonha exhibits patterns consistent with nutrient enrichment associated with urban inputs. Although the lakes differ in their dominant drivers, both systems display conditions that may increase environmental vulnerability. PCA scores show considerable variability (9.95 in AP; 7.71 in BL), suggesting stronger temporal fluctuations in AP (Table 5). In contrast, BL presents a more persistent pattern associated with nutrient dynamics (Table 5). Regarding chemical sensitivity, ASI values indicate reduced buffering conditions in both systems. Mean ASI values (−1.028 for AP; −1.095 for BL) suggest overall sensitivity, while amplitude ranges (up to 2.76) and minimum values (down to −2.56) indicate that buffering capacity may be substantially reduced during periods of intense rainfall.

These findings suggest that pH alone is insufficient to characterize system stability. Effective management should consider seasonal dynamics, with emphasis on metabolic processes during the dry season (e.g., CO_2_ and DO) and buffering capacity during the wet season (e.g., ASI and TA). This integrated approach, incorporating metabolism, buffering, nutrient dynamics, and synthetic indices, provides a comprehensive framework for assessing the geochemical status of these important water supply reservoirs.

## Conclusions

This research demonstrates that acidification in Lakes Água Preta and Bolonha is a multifaceted process, in which metabolic stress and geochemical vulnerability operate across different spatial and temporal scales. Through the application of a ‘Dual Diagnosis’ framework, both reservoirs were identified as chronically sensitive due to limited regional alkaline reserves, as indicated by mean ASI values of −1.03 (AP) and −1.10 (BL). Based on mineral saturation criteria, these indices suggest high sensitivity, with episodes of increased instability during the wet season, when minimum values reach approximately −2.57.

The dominant drivers of acidification differ between the systems:

- Lake Água Preta (AP): Primarily associated with inorganic carbon dynamics and heterotrophic respiration, exhibiting greater temporal variability, with a PCA amplitude of 9.95.
- Lake Bolonha (BL): More strongly influenced by nutrient dynamics linked to cultural eutrophication, with a PCA amplitude of 7.72.

Vertical analysis indicates that bottom waters are generally associated with conditions of lower dissolved oxygen and reduced buffering capacity. This layer acts as an important zone for biogeochemical transformations, where anoxia and CO₂ accumulation are linked to reduced ionic stability, as reflected by slightly higher ASI variability at depth (2.72) compared to the surface (2.66).

Seasonally, the systems exhibit a contrasting pattern:

- Dry season: Associated with increased metabolic activity and elevated PCA values.
- Wet season: Associated with reduced buffering capacity and lower ASI values.

These results indicate that, although rainfall may dilute certain solutes, it can also reduce ionic buffering capacity, increasing sensitivity to acidification. Consequently, pH alone is insufficient to characterize the chemical dynamics of these reservoirs.

Environmental management should therefore consider seasonal variability, with emphasis on metabolic processes during the dry season (e.g., CO_2_ and DO) and on buffering capacity during the wet season (e.g., ASI and TA). This integrated approach provides a comprehensive framework to support water quality management in the metropolitan region of Belém.

## Supplementary Information

Below is the link to the electronic supplementary material.

Supplementary file1 (DOCX 1056 KB)

## Data Availability

Data would be available by request.
