# Supplementary material for: Dual diagnosis of acidification dynamics in Amazonian urban lakes: multivariate analysis and calcite saturation index for status and vulnerability assessment
Source: Environ Monit Assess. 2026 May 27;198(6):661. doi: 10.1007/s10661-026-15475-y (PMC13216101; doi:10.1007/s10661-026-15475-y)
Supplement: Supplementary file 1 — Supplementary file1 (DOCX 1056 KB) [file 10661_2026_15475_MOESM1_ESM.docx]

**Supplementary material**

**Title:** Dual diagnosis of acidification in Amazonian urban lakes: multivariate analysis and calcite saturation index for status and vulnerability assessment.

**Authors**: Carlos Noriega^1^; Cryssia Romão^2^; Rafael Aquino^2^; Sury Monteiro^2^; Bruna Moraes^2^; Rodrigo Brito^2^; Carmen Medeiros^1^; Marcelo Rollnic^2^; Moacyr Araujo^1,3^.

**Addresses**: ^1^Department of Oceanography, Federal University of Pernambuco – UFPE, Av. Arquitetura s/n, 50740-550, Recife, Brazil; ^2^Laboratório de Pesquisa e Monitoramento Ambiental Marinho (LAPMAR), Federal University of Pará, Rua Augusto Corrêa N° 1, Belém 66075-900, PA, Brazil; ^3^Brazilian Research Network on Global Climate Change (Rede CLIMA), Av. dos Astronautas, 1758, 01227-010 São José dos Campos, SP, Brazil.

***Corresponding author**: [cnoriega.ufpe@gmail.com](mailto:cnoriega.ufpe@gmail.com); ORCid: 0000-0002-3590-6377.

**1. Bathymetry**


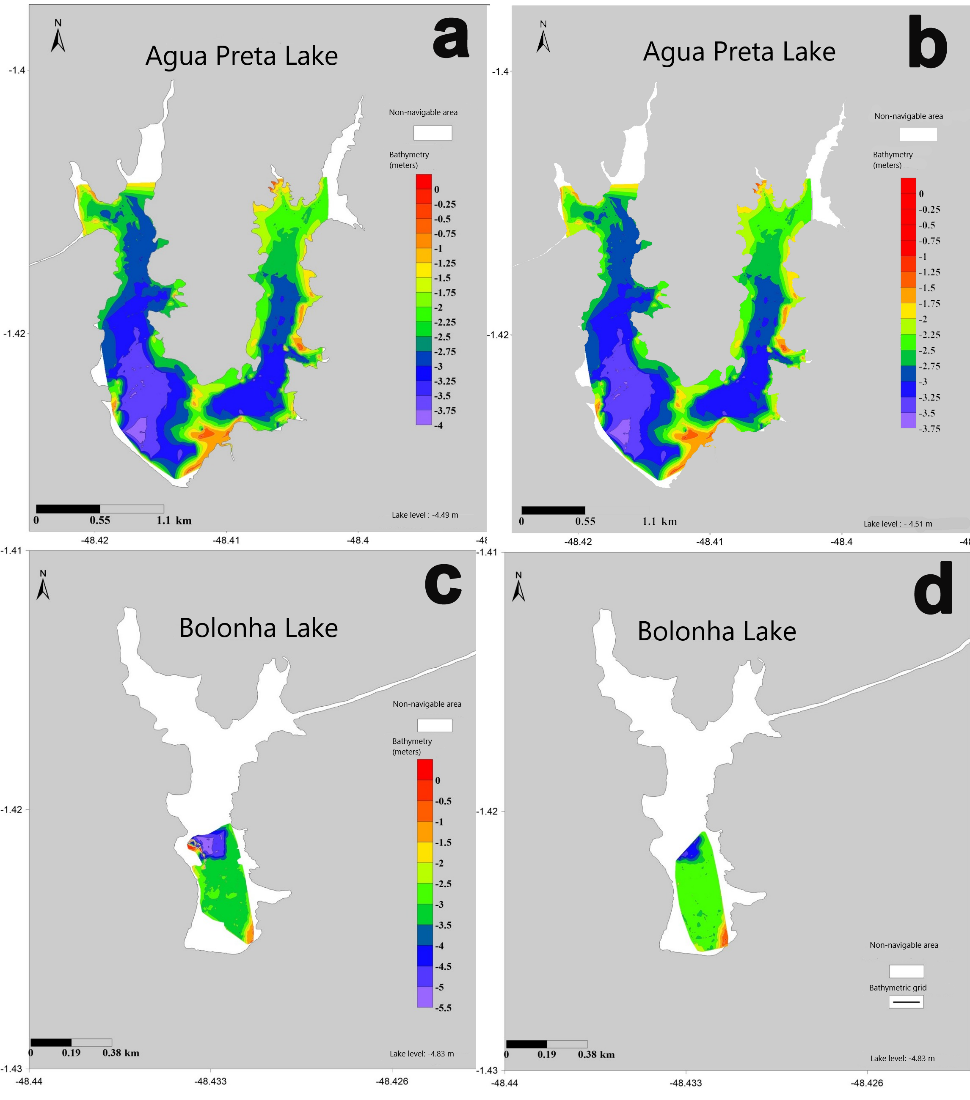


**Fig.S1**. Bathymetry of Lake Agua Preta (a-b) and Bolonha (c-d) during the dry season (a and c) and rainy season (b and d). The white area in Bolonha Lake represents an area that is not navigable due to the coverage of macrophytes.

**2. Descriptive statistics**

**Table S1**. Descriptive statistics of the physical-chemical parameters on the surface and at the bottom of lakes AP and BL. SD: Standard Deviation; CV: Coefficient of Variation; *indicates significant differences between lakes (Mann-Whitney test); * indicates significant differences between layers (Mann-Whitney test).

| **Parameter** | **Lake** | **Mean**  **Bottom** | **Mean**  **Surface** | **SD**  **Bottom** | **SD**  **Surface** | **CV**  **Bottom** | **CV**  **Surface** | **Overall**  **CV** |
| --- | --- | --- | --- | --- | --- | --- | --- | --- |
| Fe (μmol⋅L^−1^) | BL | 35.79 | 26.68 | 18.06 | 13.91 | 50.46 | 52.14 | 70.00 |
| Fe (μmol⋅L^−1^) | AP | 48.74 | 37.60 | 19.34 | 12.08 | 39.68 | 32.13 | 49.70 |
| DO (μmol⋅L^−1^) | BL | 72.82 | 131.15 | 41.56 | 63.87 | 57.07 | 48.70 | 47.60** |
| DO (μmol⋅L^−1^) | AP | 100.91 | 217.61 | 58.75 | 77.01 | 58.22 | 35.39 | 81.10** |
| pH | BL | 6.45 | 6.83 | 0.29 | 0.28 | 4.53 | 04.05 | 5.70** |
| pH | AP | 6.11 | 6.63 | 0.40 | 0.36 | 6.56 | 6.34 | 9.50** |
| Dissolved CO_2_​ (μmol⋅L^−1^) | BL | 225.29 | 185.48 | 101.45 | 75.31 | 45.03 | 40.59 | 75.60 |
| Dissolved CO_2_​ (μmol⋅L^−1^) | AP | 250.77 | 184.18 | 131.98 | 65.53 | 52.63 | 35.58 | 115.10* |
| TA (μmol⋅L^−1^) | BL | 163.67 | 163.85 | 37.10 | 34.02 | 22.67 | 20.76 | 48.20* |
| TA (μmol⋅L^−1^) | AP | 95.89 | 111.68 | 27.65 | 23.36 | 28.83 | 20.92 | 55.00* |
| NH_4_^+^ (μmol⋅L^−1^) | BL | 3.42 | 1.68 | 2.74 | 1.10 | 80.12 | 65.48 | 131.30* |
| NH_4_^+^ (μmol⋅L^−1^) | AP | 3.10 | 1.53 | 2.67 | 01.09 | 86.13 | 71.24 | 133.20* |
| PO_4_^3-^ (μmol⋅L^−1^) | BL | 0.51 | 0.21 | 0.39 | 0.18 | 76.43 | 84.05 | 74.40 |
| PO_4_^3-^ (μmol⋅L^−1^) | AP | 0.48 | 0.22 | 0.40 | 0.18 | 83.92 | 81.82 | 168.20 |
| EC (μS⋅cm^−1^) | BL | 129.54 | 124.90 | 25.17 | 21.05 | 19.43 | 16.85 | 71.00 |
| EC (μS⋅cm^−1^) | AP | 94.01 | 85.06 | 21.23 | 15.69 | 22.58 | 18.44 | 76.10 |
| TDS (g⋅L^−1^) | BL | 0.08 | 0.08 | 0.02 | 0.01 | 19.40 | 16.85 | 66.20 |
| TDS (g⋅L^−1^) | AP | 0.06 | 0.05 | 0.01 | 0.01 | 22.53 | 18.42 | 75.80 |
| Temperature (^∘^C) | BL | 29.23 | 29.62 | 1.18 | 1.16 | 04.04 | 3.92 | 2.30** |
| Temperature (^∘^C) | AP | 28.89 | 29.41 | 1.35 | 1.53 | 4.66 | 5.19 | 2.50** |
| Ca^2+^ (mg⋅L^−1^) | BL | 0.26 | 0.25 | 0.05 | 0.04 | 19.23 | 16.00 | 119.10 |
| Ca^2+^ (mg⋅L^−1^) | AP | 0.23 | 0.20 | 0.06 | 0.04 | 26.09 | 20.00 | 118.50 |
| Mg^2+^ (mg⋅L^−1^) | BL | 0.07 | 0.07 | 0.01 | 0.01 | 14.29 | 14.29 | 99.30* |
| Mg^2+^ (mg⋅L^−1^) | AP | 0.06 | 0.05 | 0.01 | 0.01 | 16.67 | 20.00 | 105.70* |
| HCO_3_^-^ (μmol⋅L^−1^) | BL | 163.67 | 163.85 | 37.10 | 34.02 | 22.67 | 20.76 | 48.10* |
| HCO_3_^-^ (μmol⋅L^−1^) | AP | 95.89 | 111.68 | 27.65 | 23.36 | 28.83 | 20.92 | 54.90* |
| NO_3_^-^ (μmol⋅L^−1^) | BL | 1.86 | 1.88 | 0.54 | 0.60 | 29.07 | 31.78 | 89.70* |
| NO_3_^-^ (μmol⋅L^−1^) | AP | 1.25 | 1.39 | 0.72 | 0.76 | 57.60 | 54.68 | 84.60* |
| NO_2_^-^ (μmol⋅L^−1^) | BL | 0.02 | 0.02 | 0.01 | 0.01 | 70.00 | 65.00 | 68.10* |
| NO_2_^-^ (μmol⋅L^−1^) | AP | 0.02 | 0.02 | 0.01 | 0.01 | 45.00 | 40.00 | 86.80* |
| SiO_2_^-^ (μmol⋅L^−1^) | BL | 39.85 | 39.38 | 25.10 | 25.43 | 63.00 | 64.59 | 82.20* |
| SiO_2_^-^ (μmol⋅L^−1^) | AP | 45.01 | 48.06 | 29.50 | 29.98 | 65.54 | 62.38 | 89.70* |


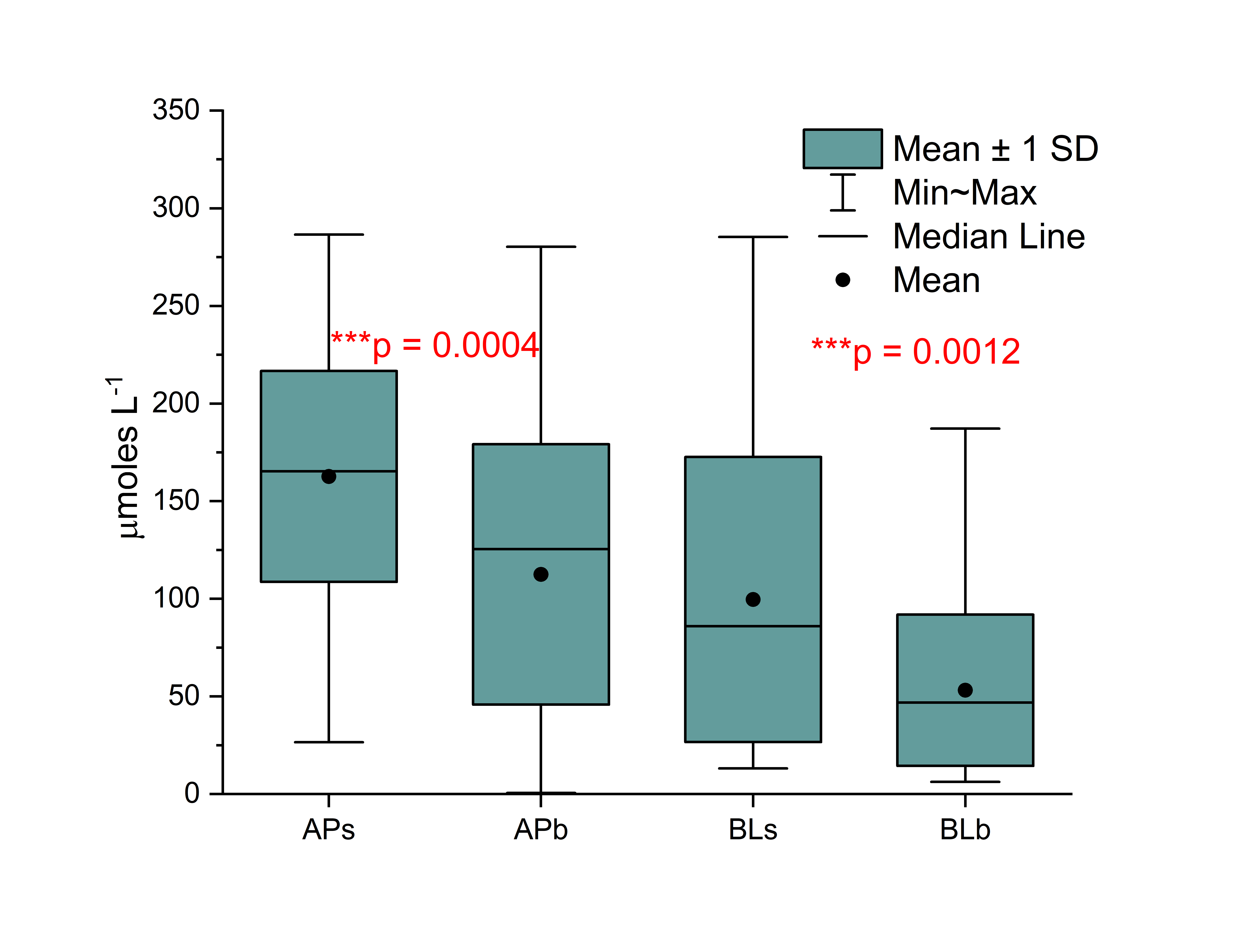


**Fig. S2**. Vertical distribution of Dissolved Oxygen (DO) in Agua Preta and Bolonha lakes. Surface and bottom layers represent the monthly time series. Asterisks indicate significant differences according to the Mann-Whitney U test (*** p < 0.001). APs: Agua Preta surface; APb: Agua Preta bottom; BLs: Bolonha surface; BLb: Bolonha bottom.

**3. Principal Component Analysis (PCA)**

**Table S2**. Variance explained by component for parameters associated with the physical-chemical study in the Agua Preta and Bolonha lakes.

| **Lake** | **Component** | **Explained Variance (%)** |
| --- | --- | --- |
| **Água Preta (AP)** | PC1 | 21.05% |
|  | PC2 | 14.16% |
|  | **Total (PC1 + PC2)** | 35.21% |
| **Bolonha (BL)** | PC1 | 23.87% |
|  | PC2 | 20.43% |
|  | **Total (PC1 + PC2)** | 44.30% |

**Table S3**. Factor loadings of the first two components in PCA for the physical-chemical parameters analyzed at the surface of Lake Agua Preta. Values in bold indicate higher factor loadings of a parameter in the components.

| **Parameter** | **PC1** | **PC2** |
| --- | --- | --- |
| Dissolved CO_2_ | **0.508** | -0.011 |
| pH | **-0.442** | 0.004 |
| TA | **0.446** | 0.129 |
| Dissolved Oxygen-DO | **-0.269** | -0.095 |
| Fe | **0.266** | -0.164 |
| EC | 0.097 | **0.388** |
| NO_3_^-^ | 0.183 | **-0.473** |
| Mg^2+^ | -0.009 | **0.347** |
| NO_2_^-^ | 0.123 | **0.334** |
| PO_4_^3-^ | 0.120 | **0.298** |
| Temperature | -0.250 | **0.267** |
| NH_4_^+^ | 0.154 | **-0.240** |
| SiO_2_^-^ | **0.209** | 0.165 |
| Ca^2+^ | -0.040 | **-0.309** |

**Table S4**. Factor loadings of the first two components in PCA for the physical-chemical parameters analyzed at the bottom of Lake Agua Preta. Values in bold indicate higher factor loadings of a parameter in the components.

| **Parameter** | **PC1** | **PC2** |
| --- | --- | --- |
| pH | **0.426** | -0.073 |
| Dissolved Oxygen-DO | **0.382** | -0.086 |
| Temperature | **0.361** | -0.160 |
| Dissolved CO_2_ | **-0.332** | 0.309 |
| NO_3_^-^ | **-0.351** | 0.126 |
| Mg^2+^ | **0.324** | 0.294 |
| EC | 0.227 | **0.235** |
| PO_4_^3-^ | 0.240 | **0.507** |
| NO_2_^-^ | 0.149 | **0.361** |
| TA | 0.006 | **0.427** |
| NH_4_^+^ | -0.108 | **0.288** |
| Fe | **-0.223** | 0.118 |
| Ca^2+^ | **-0.109** | 0.014 |
| SiO_2_^-^ | 0.002 | **0.203** |

**Table S5**. Factor loadings of the first two components in PCA for the physical-chemical parameters analyzed at the surface of Lake Bolonha. Values in bold indicate higher factor loadings of a parameter in the components.

| **Parameter** | **PC1** | **PC2** |
| --- | --- | --- |
| NO_2_^-^ | **0.411** | -0.134 |
| EC | **0.408** | -0.159 |
| Fe | **-0.325** | -0.268 |
| NO_3_^-^ | **-0.318** | 0.187 |
| Dissolved CO_2_ | **-0.339** | -0.274 |
| PO_4_^3-^ | 0.310 | **-0.340** |
| Mg^2+^ | **0.230** | 0.001 |
| Temperature | **0.227** | 0.166 |
| pH | **0.244** | 0.020 |
| Dissolved Oxygen-DO | 0.077 | **0.399** |
| TA | -0.208 | **-0.437** |
| SiO_2_^-^ | -0.016 | **-0.356** |
| Ca^2+^ | -0.086 | **0.317** |
| NH_4_^+^ | -0.162 | **0.232** |

**Table S6**. Factor loadings of the first two components in PCA for the physical-chemical parameters analyzed at the bottom of Lake Bolonha. Values in bold indicate higher factor loadings of a parameter in the components.

| **Parameter** | **PC1** | **PC2** |
| --- | --- | --- |
| NO_2_^-^ | **-0.415** | -0.133 |
| PO_4_^3-^ | **-0.396** | -0.122 |
| EC | **-0.352** | -0.205 |
| TA | **-0.346** | 0.321 |
| Temperature | **-0.340** | -0.024 |
| Mg^2+^ | **-0.311** | 0.169 |
| NO_3_^-^ | **0.304** | 0.297 |
| Dissolved CO_2_ | -0.258 | **0.453** |
| NH_4_^+^ | **0.186** | -0.087 |
| Ca^2+^ | 0.102 | **0.181** |
| Fe | 0.056 | **0.479** |
| Dissolved Oxygen-DO | 0.001 | **-0.317** |
| pH | 0.086 | **-0.328** |
| SiO_2_^-^ | -0.038 | **-0.147** |

**4. Temporal analysis of physical-chemical parameters**


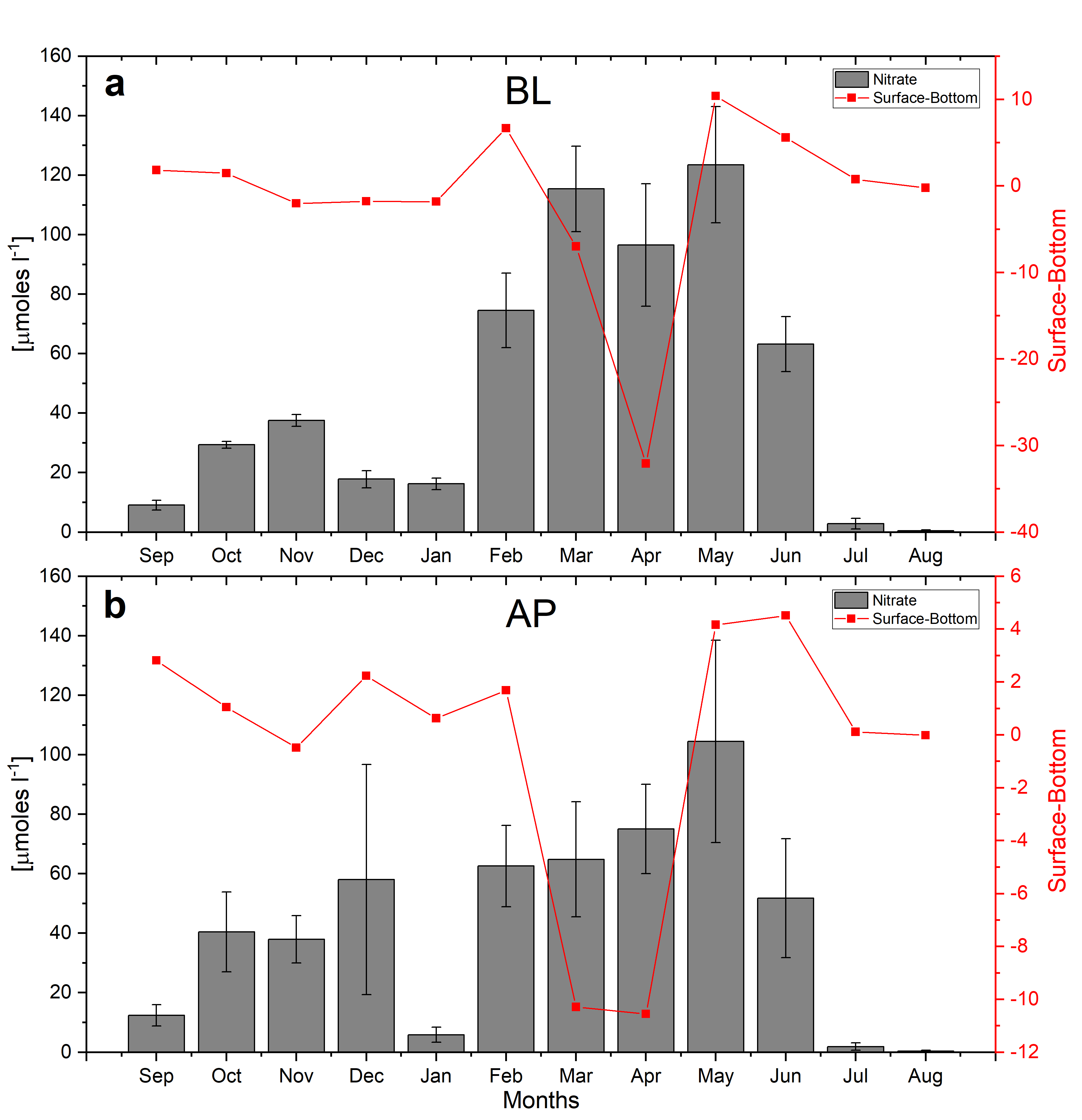


**Fig. S3.** Time series of nitrate (NO_3_^-^) in BL (**a**) and AP (**b**) throughout an annual cycle. Red lines indicate the surface-to-bottom difference for each month. Error bars (black lines) represent the monthly standard deviation.


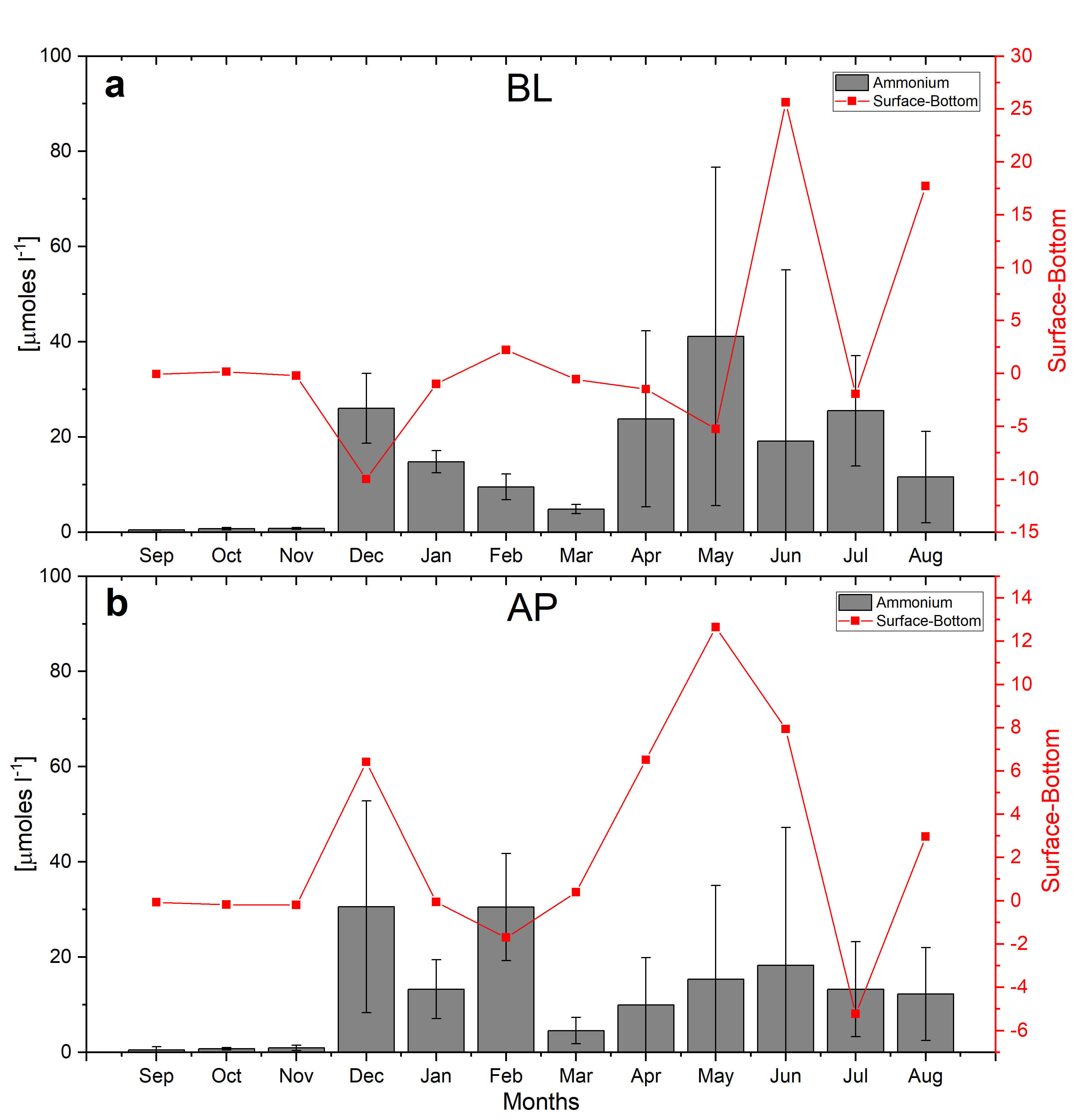


**Fig. S4.** Time series of ammonium (NH4^+^) in BL (**a**) and AP (**b**) throughout an annual cycle. Red lines indicate the surface-to-bottom difference for each month. Error bars (black lines) represent the monthly standard deviation.
